# Lanthanoid-containing polyoxometalate nanocatalysts in the synthesis of bioactive isatin-based compounds

**DOI:** 10.1038/s41598-022-16384-z

**Published:** 2022-07-14

**Authors:** Mansoureh Daraie, Masoud Mirzaei, Maryam Bazargan, Vadjiheh Sadat Amiri, Bita Abdolahi Sanati, Majid M. Heravi

**Affiliations:** 1 Department of Chemistry, School of Sciences, Alzahra University, Vanak, Tehran, Iran; 2 Department of Chemistry, Faculty of Science, Ferdowsi University of Mashhad, 9177948974 Mashhad, Iran

**Keywords:** Catalysis, Inorganic chemistry, Organic chemistry

## Abstract

Lanthanoid-containing polyoxometalates (Ln-POMs) have been developed as effective and robust catalysts due to their Lewis acid–base active sites including the oxygen-enriched surfaces of POM and the unique 4f. electron configuration of Ln. As an extension of our interest in Ln-POMs, a series of as-synthesized nanocatalysts K_15_[Ln(BW_11_O_39_)_2_] (**Ln-B**_**2**_**W**_**22**_, Ln = La, Ce, Nd, Sm, Gd, and Er) synthesized and fully characterized using different techniques. The Ln^3+^ ion with a big ionic radius was chosen as the Lewis acid center which is sandwiched by two mono-lacunary Keggin [BW_11_O_39_]^9−^ units to form Ln-containing sandwiched type cluster. Consequently, the catalytic activity of nanocatalysts with different Ln was examined in the synthesis of bioactive isatin derivatives and compared under the same optimized reaction conditions in terms of yields of obtained products, indicating the superiority of the nano-**Gd-B**_**2**_**W**_**22**_ in the aforementioned simple one-pot reaction. The effects of different dosages of nanocatalyst, type of solvent, reaction time, and reaction temperature in this catalytic system were investigated and the best results were obtained in the presence of 10 mol% of nano-**Gd-B**_**2**_**W**_**22**_ in water for 12 min at the reflux condition.

## Introduction

The term “spiro” in organic chemistry was firstly defined by Von Baeyer in the late 1890s. This term is used when two hydrocarbon rings are assembled on a shared carbon atom which is named the spiro carbon atom. Presently, spiro organic structures are considered in designing new pharmaceuticals. The special biological and conformational characteristics with the complexity and rigidity properties of the spiro compounds, make them good chiral candidates in drug discovery^1–4^.

**Figure 1:**
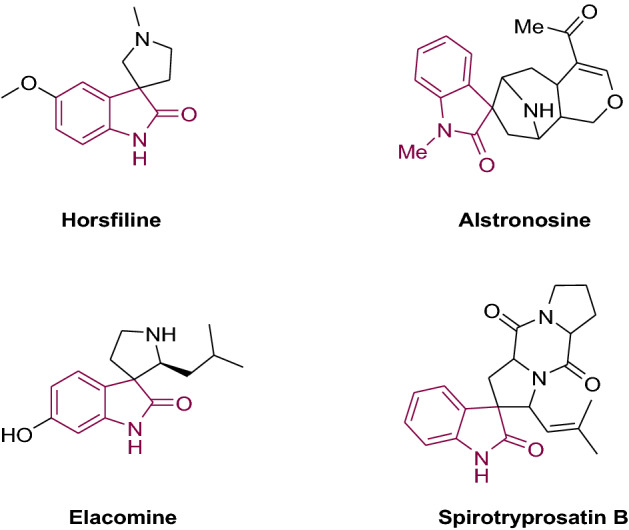
Selected spirooxindole natural products.

Spirooxindole core is one of the most popular spiro compounds found in the structure of many alkaloids, bioactive synthetic compounds, and pharmaceuticals (Fig. 1)^5^. Spirooxindoles have shown various biological activities, including promising anticancer^6^, antimicrobial^7^, antiviral, antioxidant, anti-inflammatory, antileishmanial, and antiplasmodial agents^8,9^. Moreover, some spirooxindole-based compounds have been developed as inhibitors of microtubule assembly, such as spirotryprostatin A, alstonisine, and ptropodind. According to the importance of spirooxindoles in drug discovery, many researches have been directed to find new efficient synthetic routes furnishing molecules containing this core^10,11^.

Polyoxometalates (POMs), known as inorganic ligands, are discrete, anionic metal-oxide clusters of group V or VI transition metals in their highest oxidation state and exhibit a great diversity of sizes, nuclearities, and shapes^12–14^. POMs benefit from interesting structural skeletons including protons (Brönsted acids, can promote acid‐catalyzed reactions), oxygen atoms (with a high negative charge can be used in base‐catalyzed reactions), and metal ions with unoccupied orbitals (Lewis acids)^15^. The motivation for choosing POMs comes not only from their intriguing structural diversity, but also they contain several potential applications in many fields such as sorbent^16,17^ catalysis^18–20^, magnetic^21^, optical materials sensitive devices^22^, electro/photochromic systems^23^, sensors^24^ and medicine^25^. Lacunary POMs are defect derivatives of saturated ones, including one or more vacant sites such as mono-lacunary, di, or tri-lacunary structures^26^. The most common lacunary POMs are derivatives of the Keggin and Wells–Dawson ions, resulting frequently in sandwich-type clusters^27,28^. Totally, structural vacancies in the lacunary POMs lead to enhance surface reactivity, therefore, they can be substituted by metals with strong Lewis acidity, such as lanthanoids or transition metals like zirconium to generate Lewis acid catalysts^29,30^.

**Figure 2:**
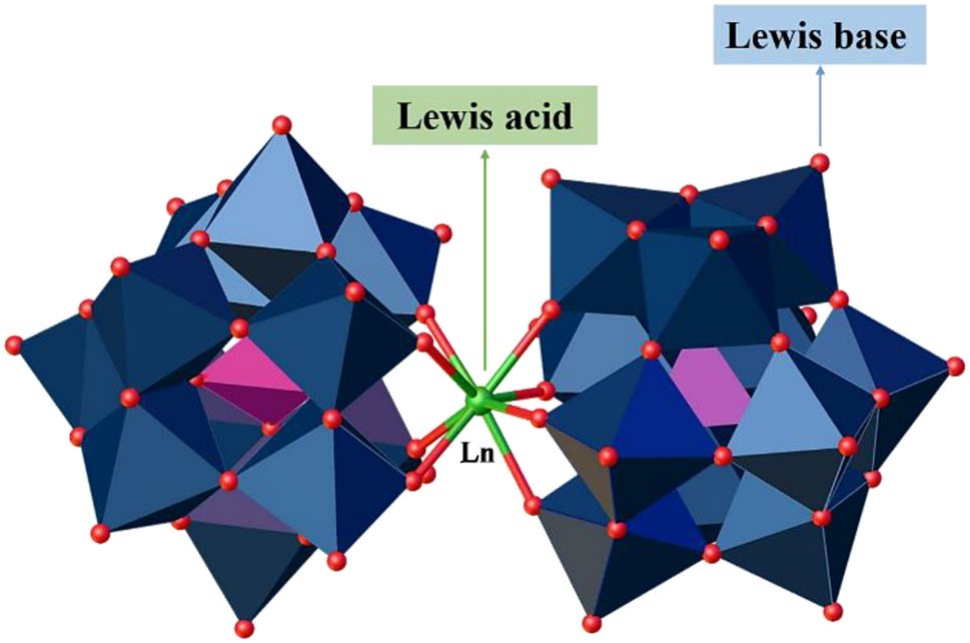
The structure of **Ln-B**_**2**_**W**_**22**_ nanocatalyst (Color code: W, dark blue; Ln, grey; O, red; B, purple). Reproduced from ref^40^ with permission.

Lanthanoid-containing polyoxometalates (Ln-POMs), specially constructed from lacunary Keggin anions are structurally rigid clusters (Fig. 2) and showed higher stability, and have Lewis acid–base active sites compared with naked POMs. Also, a synergistic combination between the Ln and POM within one molecular structure can enhance their potential application in many fields such as luminescence, magnetism, and catalysis^31,32^. Furthermore, due to their easy synthetic procedure and their robustness nature in the solid and solution, they can be also used in acid/base-catalyzed reactions for laboratory research purposes and industrial applications. Although there are several examples of isatin-based compounds synthesized using POMs or POMs-based composites^33–36^, Lewis acid catalysts containing Ln-POMs have rarely been studied for them.

Herein, we have successfully synthesized a series of isostructural α-Keggin borotungstate dimers with Ln cations, [Ln(BW_11_O_39_)_2_]^15−^ (**Ln-B**_**2**_**W**_**22**_, Ln = La, Ce, Nd, Sm, Gd, and Er). Then, the related nanocatalysts were prepared by the top-down approach using the ultrasonic technique. In continuation of our efforts towards advancing synthetic methods to achieve spirooxindoles, in this research, we want to introduce a highly efficient, environmentally benign, and simple one-pot method for the nano-**Gd-B**_**2**_**W**_**22**_-catalyzed synthesis of bioactive spirooxindole derivatives^37–39^.

## Experimental

### Chemicals and materials

The chemical compounds were purchased from Merck (Darmstadt, Germany, www.merckmillipore.com) and Sigma-Aldrich (St. Louis, MO, USA, www.sigmaaldrich.com) and used with no crystallization or purification.

### Instrumentation

Electrothermal 9200 apparatus was employed to determine the melting point of products. Bruker Tensor 27 FT-IR spectrometer (400–4000 cm^–1^ region) was used to detect absorbance bands of organic products using a KBr disk containing the compounds. ^1^H NMR, ^13^C NMR spectra were recorded on a Bruker AQS 400-AVANCE spectrometer at 400 and 100 MHz, respectively, using TMS as an internal standard (DMSO solution). Also, the infrared spectra of catalysts were recorded in the range of 4000–400 cm^–1^ on a Thermo Nicolet/AVATAR 370 Elemental analysis (CHN) was performed using a Thermo Finnigan Flash EA 1112 microanalyzer. Metal content was measured by the Spectro Arcos ICP-OES spectrometer model 76004555 using in the range of 130–770 nm for ICP spectra. Powder X-ray diffraction (PXRD) data were collected on ASENWARE/AW-XDM300 X-ray powder diffractometer using Cu Kα (λ = 1.54184 Å) radiation at room temperature with the scan range 2θ = 3 to 40° and step size of 0.05° and step time of 1 s. The scanning electron microscope (SEM) analysis, EDS, and EDS mapping were recorded using LEO-1450 VP at an acceleration voltage of 10.00 kV and resolution of about 500 nm (Zeiss, Germany).

### Preparation of catalysts

The mono-lacunary Keggin K_9_[BW_11_O_39_]·13H_2_O was synthesized according to a literature method and identified by FT-IR and elemental analysis^41^. Then, mono-lacunary Keggin can be stabilized by lanthanide centers in solution and in the solid-state to form sandwich-type polyoxometalates K_15_[Ln(BW_11_O_39_)_2_]·nH_2_O (**Ln-B**_**2**_**W**_**22**_, Ln = La, Ce, Nd, Sm, Gd, and Er)^40,42^.

General synthetic procedure for catalysts. A mixture of lanthanoid nitrate (0.085 mmol) and K_9_[BW_11_O_39_]·13H_2_O (0.155 mmol) in 20 mL of KCl (1 M) was stirred for 10 min in air and then the pH was adjusted to 5.0 by dropwise addition of 0.1 M KOH. The resulted mixture was stirred for a further 40 min at 50 °C. Pure crystals of the catalysts were obtained by slow evaporation of the solvent after several days.

Synthesis of nanocatalysts. The mixture solution of Ethanol (10 mL), water (15 mL), and **Ln-B**_**2**_**W**_**22**_ crystals (0.03 g) were subjected to ultrasonication (150 W). After 20 min, nanocatalysts were collected by the centrifuge and then washed with cold water (3 × 5 mL) under vacuum. FT-IR spectra (KBr pellet, cm^−1^) of nano-**Ln-B**_**2**_**W**_**22**_ were consistent with their spectra before doing the nano procedure (Fig. S2).

**Figure 3:**
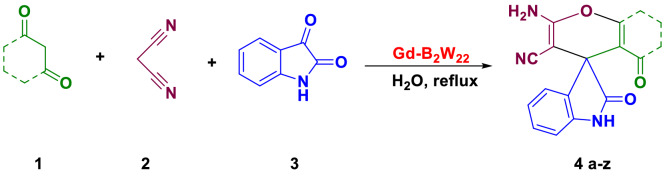
Synthesis of spiro-2-amino-4H-pryans.

General procedure for the synthesis of spiro-2-amino-4H-pryans. A combination of 1,3-diketone, carbonyl compound (either isatin or acenaphtoquinone), α-cyano compound (either malononitrile or ethyl cyanoactetate), and **Gd-B**_**2**_**W**_**22**_ was stirred in water at ambient temperature until the complete formation of the product was traced by TLC (Fig. 3). Then, the crude product was filtered, washed with water and dissolved in hot ethanol for crystallization. Furthermore, all products were characterized and analyzed by melting points and FT-IR spectra, and the results were compared with those reported in the literature to prove the formation of target products.

**Figure 4:**
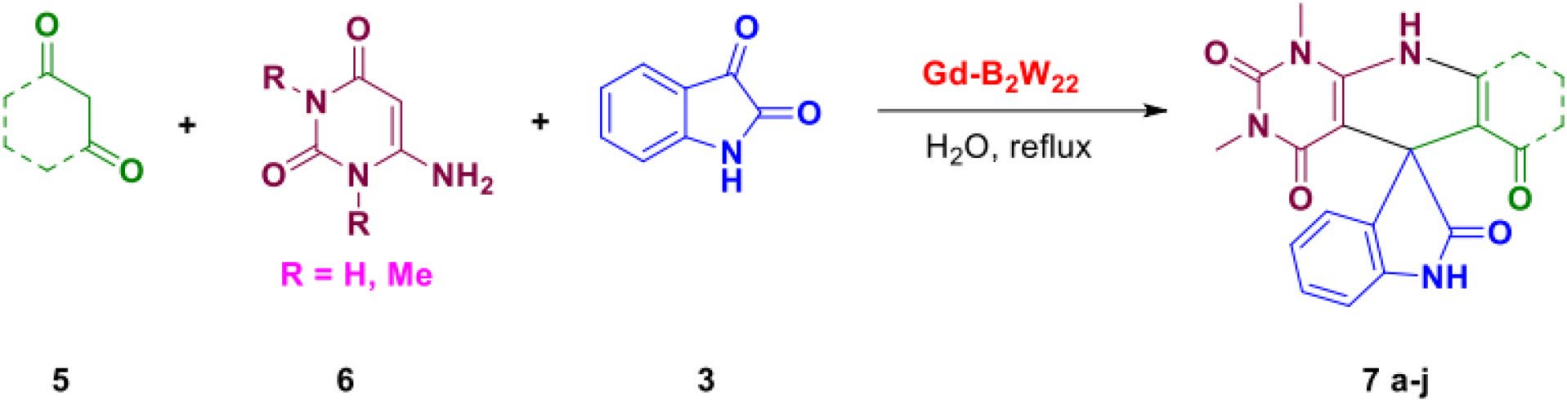
Synthesis of uracil-fused spirooxindoles.

General procedure for the synthesis of uracil fused spirooxindoles. A combination of isatin, uracil derivative (either 1,3-dimethyl-6-aminouracil or 6-aminouracil), 1,3-diketone compounds, and **Gd-B**_**2**_**W**_**22**_ was stirred in refluxing water for 8–26 min (Fig. 4). Then, the mixture was filtered, washed well with water and dried at 80 °C. The product was recrystallized for further purification in hot ethanol. All products were characterized by melting point and the characterizations were compared with that of in literature.

**Figure 5:**
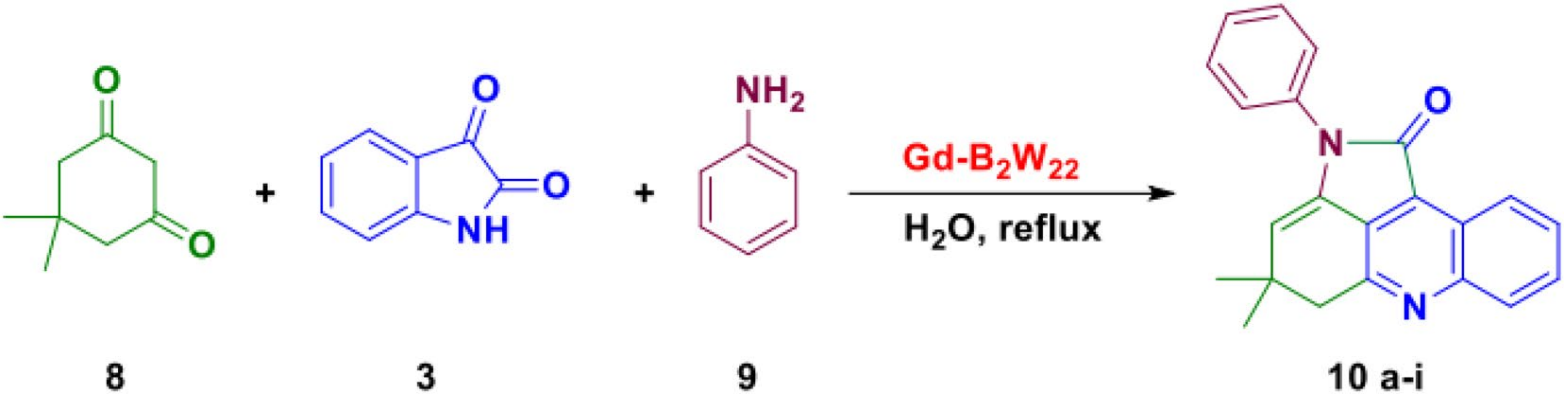
Synthesis of pyrroloacridine derivatives.

Synthesis of pyrroloacridine derivatives. A mixture of isatin, aniline, dimedone and nanocatalyst was refluxed in water for an appropriate time (Fig. 5). By the completion of the reaction, the mixture was cooled down and filtered. Then the crude product was washed well with hot water, and finally crystallized in hot EtOH. The characterization data of products were compared with that published in the literature.

### Characterization data

Spectral data for catalyst:

- **La-B**_**2**_**W**_**22**_. Colorless needle-like crystals in 52.6% yield (based on W). Anal. Calcd. for H_52_O_104_LaK_15_B_2_W_22_: K, 9.01; W, 65.2; La, 2.13; B, 0.33; H, 0.81%. Found: K, 9.37; W, 63.66; La, 2.38; B, 0.31; H, 0.77%. FT-IR (KBr pellet, cm^−1^): 3451, 1616, 1254, 997, 948, 887, 832, 777, 521.
- **Ce-B**_**2**_**W**_**22**_. Orange needle-like crystals in 55% yield (based on W). Anal. Calcd. for H_58_O_107_CeK_15_B_2_W_22_: K, 8.94; W, 61.62; Ce, 2.13; B, 0.33; H, 0.89%. Found: K, 9.21; W, 60.75; Ce, 2.09; B, 0.31; H, 0.92%. FT-IR (KBr pellet, cm^−1^): 3446, 1616, 1252, 996, 947, 887, 831, 777, 522.
- **Nd-B**_**2**_**W**_**22**_. Light purple needle-like crystals in 49% yield (based on W). Anal. Calcd. for H_50_O_103_NdK_15_B_2_W_22_: K, 9.03; W, 62.27; Nd, 2.22; B, 0.33; H, 0.78%. Found: K, 9.32; W, 63.41; Nd, 2.18; B, 0.31; H, 0.81%. FT-IR (KBr pellet, cm^−1^): 3441, 1617, 1243, 996, 984, 885, 832, 777, 520.
- **Sm-B**_**2**_**W**_**22**_. Colorless needle-like crystals in 62% yield (based on W). Anal. Calcd. for H_50_O_103_SmK_15_B_2_W_22_: K, 9.02; W, 62.21; Sm, 2.31; B, 0.33; H, 0.78%. Found: K, 9.06; W, 63.41; Sm, 2.21; B, 0.31; H, 0.76%. FT-IR (KBr pellet, cm^−1^): 3438, 2917, 1611, 1253, 1000, 494, 884, 831, 778, 519.
- **Gd-B**_**2**_**W**_**22**_. Colorless needle-like crystals in 65% yield (based on W). Anal. Calcd. for H_60_O_108_GdK_15_B_2_W_22_: K, 8.89; W, 61.30; Gd, 2.38; B, 0.33; H, 0.92%. Found: K, 9.01; W, 61.45; Gd, 2.31; B, 0.31; H, 98%. FT-IR (KBr pellet, cm^−1^): 3471, 1611, 1253, 1000, 948, 883, 832, 799, 517.
- **Er-B**_**2**_**W**_**22**_. Colorless needle-like crystals in 53% yield (based on W). Anal. Calcd. for H_52_O_104_ErK_15_B_2_W_22_: K, 8.97; W, 61.88; Er, 2.56; B, 0.33; H, 0.80%. Found: K, 9.03; W, 61.51; Er, 2.51; B, 0.32; H, 0.91%. FT-IR (KBr pellet, cm^−1^): 3428, 1621, 1258, 997, 948, 886, 835, 780, 522.

## Results and discussion

### Characterization of catalysts

**Figure 6:**
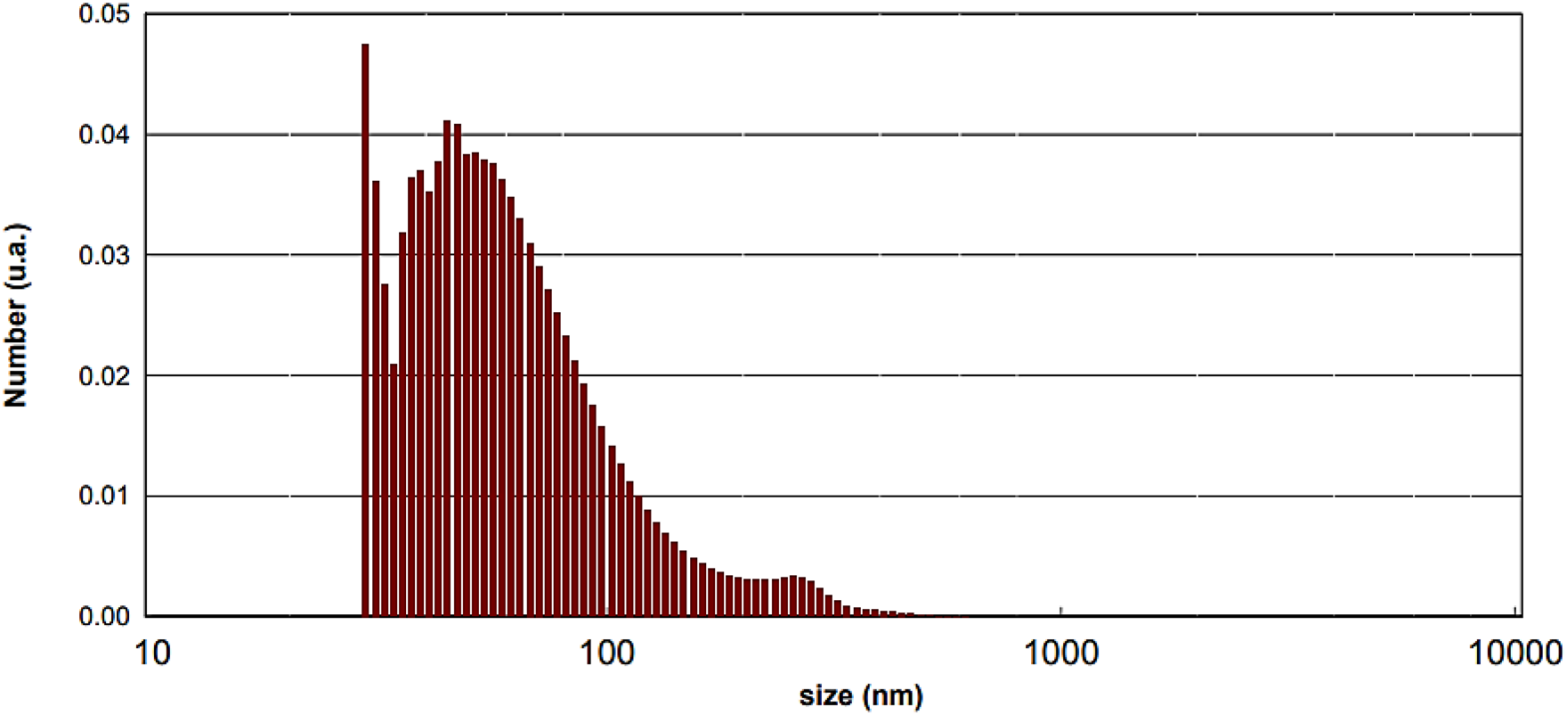
Particle size distribution histogram of nano-**Gd-B**_**2**_**W**_**22**_.

Firstly, six lanthanoid-containing polyoxometalate K_15_[Ln(BW_11_O_39_)_2_] (**Ln-B**_**2**_**W**_**22**_, Ln = La, Ce, Nd, Sm, Gd, and Er) crystals (microscopic size) of this study were obtained by reaction of the lanthanoid ion with the mono-lacunary Keggin [BW_11_O_39_]^9−^ at pH 5 (Figs. 2 and S3). Next, the above crystals were solved and subjected to ultrasonication and then nanocatalysts were collected by the centrifuge (top-down approach). The distribution histograms reveal that the average particle size of catalysts is less than 100 nm upon 20 min of sonication (Fig. 6). Particle size distribution histogram of other nanocatalysts are given in the Supplementary Figs. S4–S8.

**Figure 7:**
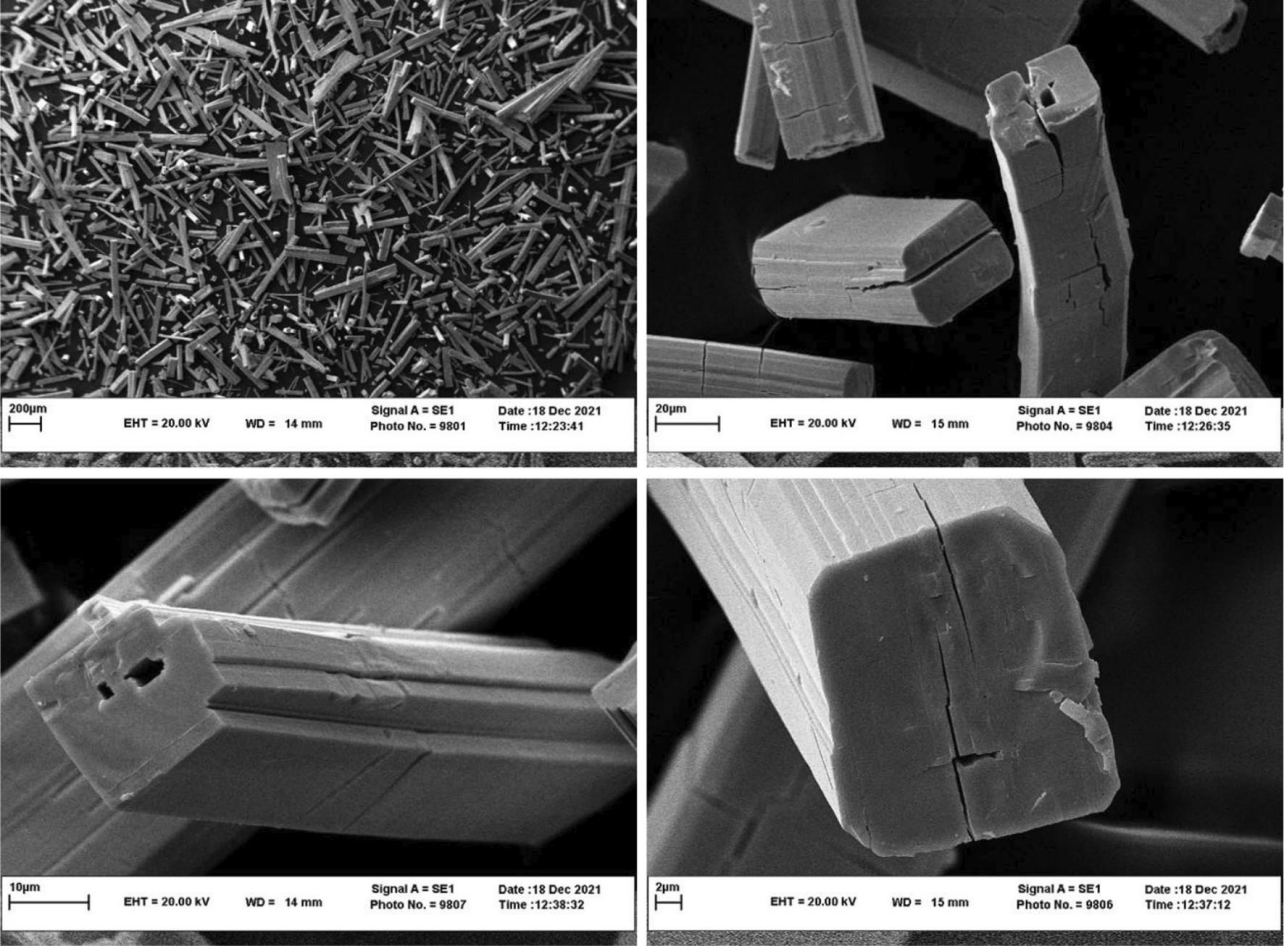
SEM images of nano-Gd-B_2_W_22_.

**Figure 8:**
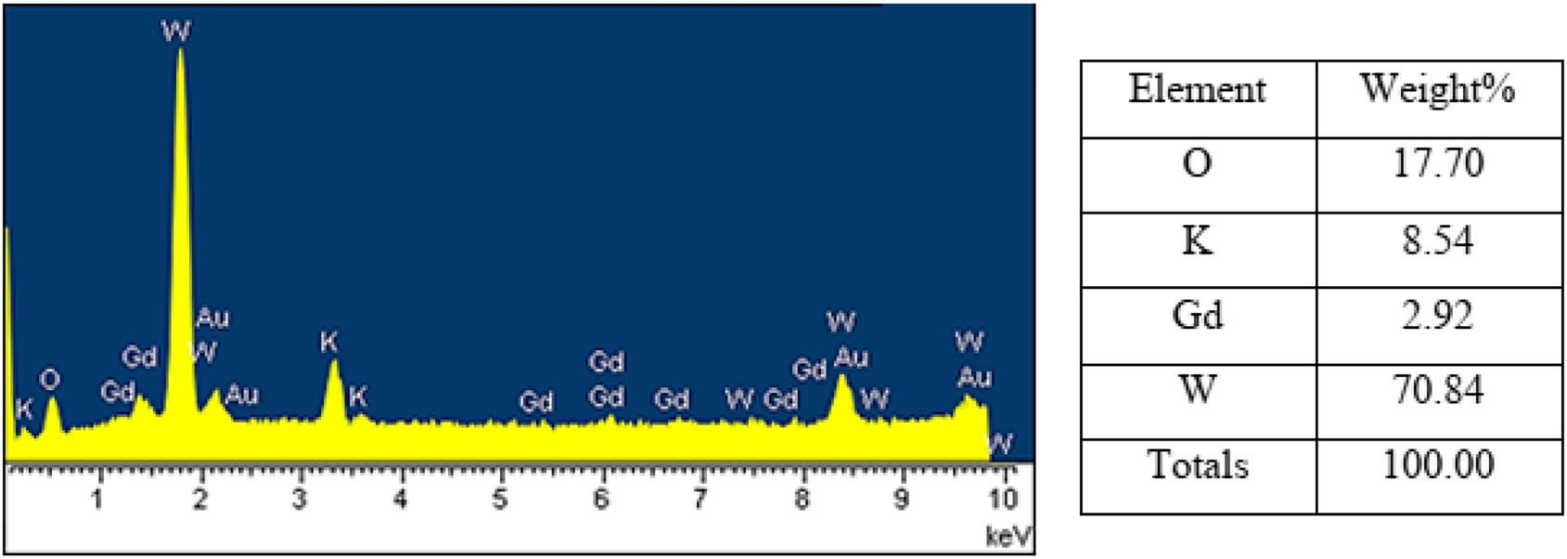
EDS spectrum of nano-Gd-B_2_W_22_.

Also, the SEM showed that the dominant morphology for nanocatalysts is rod-like (Fig. 7). Furthermore, the presence of O, K, Gd, and W in the nanocatalysts is confirmed by the EDS spectrum (Fig. 8). SEM images of **La-B**_**2**_**W**_**22**_ and EDS spectra of other nanocatalysts are given in Supplementary Information (Figs. S9–S14).

**Figure 9:**
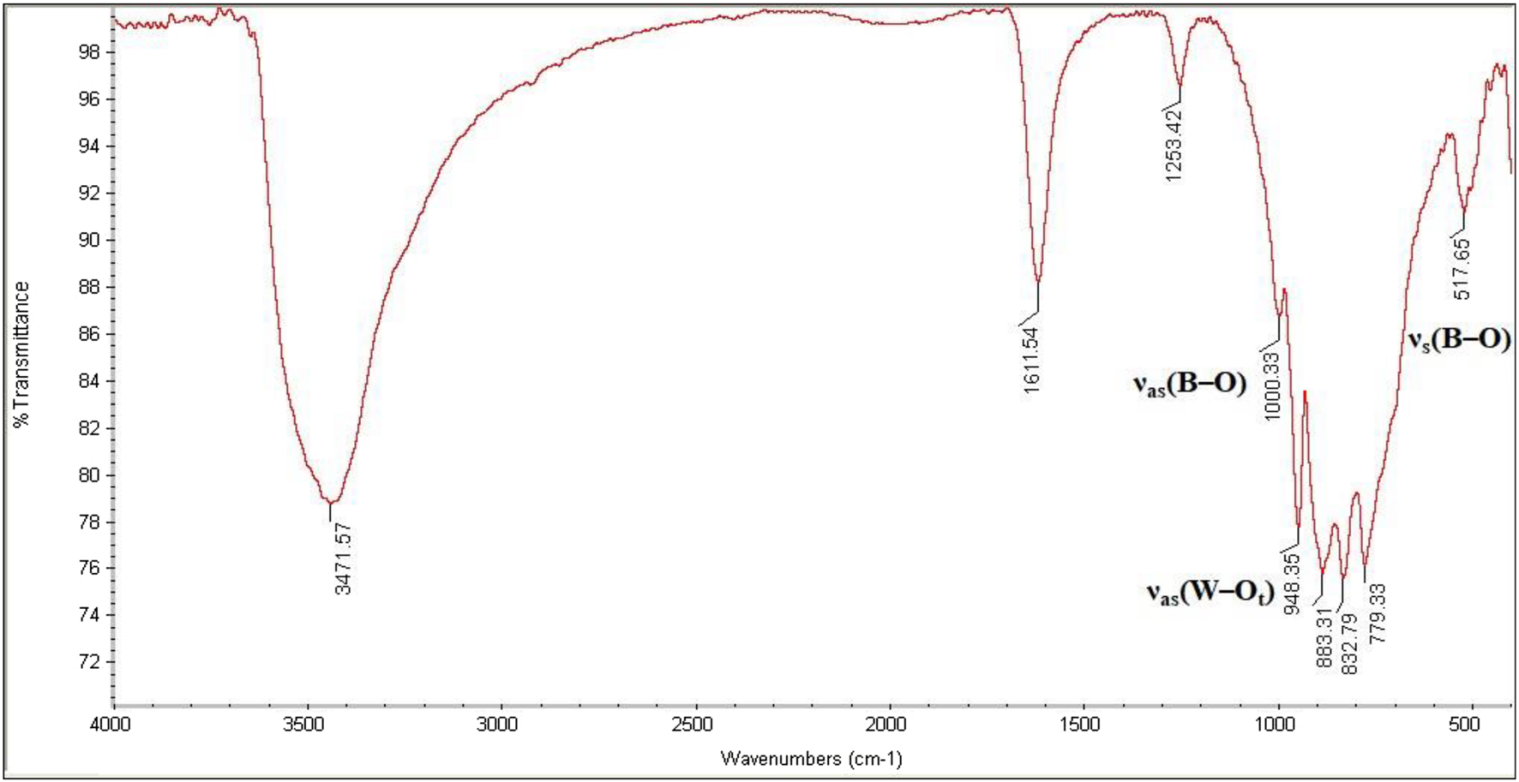
FTIR spectrum of nano-**Gd-B**_**2**_**W**_**22**_.

It is important to note that infrared spectroscopy is frequently employed technique for the characterization of POMs due to their characteristic metal–oxygen stretching vibrations that occur in the region between 400 and 1000 cm^−1^ which is known as the fingerprint region for the POMs. As shown in Figs. S1, S2, and Table 1, the overlaid IR spectra strongly suggest the same structural family for all crystalline and nano compounds. Also, the IR spectra of catalysts present a similar vibration pattern with the mono-lacunary Keggin [BW_11_O_39_]^9−^, confirming the presence of the [BW_11_O_39_]^9−^ moiety in all compounds. Briefly, nano-**Gd-B**_**2**_**W**_**22**_ showed the absorption bands at 1610 and 3471 cm^−1^ which attributed to the water molecules. The band at around 1250 cm^−1^ is attributed for bending frequencies of O–B–O. Also, characteristic bands of the terminal oxygens ν_as_(W–O_t_) at 948 cm^−1^ showed a red shift in comparison with the naked [BW_11_O_39_]^9−^ (995 cm^−1^) that indicated [BW_11_O_39_]^9−^ anions coordinated to Ln^3+^ center (Fig. 9).

**Table 1:** Representation of important absorption bands (cm^-1^) for K_15_[Ln(BW_11_O_39_)_2_] (Ln = La, Ce, Nd, Sm, Gd, and Er) and naked [BW_11_O_39_]^9−^ for comparison.

Compound	ν_as_(B–O_a_)	ν_s_(B–O_a_)	ν(W–O_a_)	ν_as_(W–O_t_)	ν_as_(W–O_b_) and ν_as_(W–O_c_)
La-B_2_W_22_	997	521	887	948	832, 777
Ce-B_2_W_22_	996	522	887	947	831, 777
Nd-B_2_W_22_	996	520	885	948	832, 777
Sm-B_2_W_22_	1000	519	884	949	831, 778
Gd-B_2_W_22_	1000	517	883	948	832, 779
Er-B_2_W_22_	997	522	886	948	835, 780
Naked BW_11_	995	515	889	954	836, 753

Also, the powder XRD pattern of the catalysts appears at around 9–10° for a 2θ value (similar to other mono-lacunary Keggin anions)^43^ (Supplementary Fig. S15).

### Catalytic activity

Ensuing this research, the catalytic activity of nano-**Ln-B**_**2**_**W**_**22**_ catalysts was tested in the model three-component reaction of isatin, malononitrile, and dimedone. To achieve eco-friendly optimized conditions, various factors were investigated. Initially, acidic catalysts including SSA, p-TSA, H_3_PW_12_O_40_, Lanthanum nitrate, K_9_[BW_11_O_39_]·13H_2_O and ZnO were chosen to compare the results obtained by catalysts (Table 2). The **Gd-B**_**2**_**W**_**22**_ nanocatalyst was selected for further tests. Next, the effect of solvent was studied by running the model reaction in polar and non-polar solvents. Finally, the amount of catalyst was optimized to achieve the highest amount of product. The reaction was also repeated with no catalyst furnishing trace amount of product. That’s while in the presence of 10 mol% of nanocatalyst, the target product was obtained in 96%. Therefore, ensuring by the effect of a catalyst in this reaction, the generalization was accomplished in water, in the presence of 10 mol% nano-**Gd-B**_**2**_**W**_**22**_ at reflux condition. It is important to note that the Lewis acidity (Z/r^3^; Z = charge and r = ionic radius) of lanthanoids decreases with an increase in the ionic radii^44^. However, among the **Ln-B**_**2**_**W**_**22**_ (Ln = La, Ce, Nd, Sm, Gd, and Er) catalysts examined, **Gd-B**_**2**_**W**_**22**_ showed better catalytic performance because by reducing the size from Gd to Er, the Er center was sterically hindered by two BW_11_ ligands and its Lewis acid site is not well accessible.

**Table 2:** Optimization of the reaction conditions.

Entry	Catalyst/amount (mol%)	Solvent	Temp. (°C)		Time (min)	Yield (%)
1	–	H_2_O		Reflux	70	20
2	Silica sulfuric acid	H_2_O		Reflux	50	80
3	p-Toluenesulfonic acid	H_2_O		Reflux	45	82
4	H_3_PW_12_O_40_	H_2_O		Reflux	40	88
5	Lanthanum nitrate	H_2_O		Reflux	25	91
6	K_9_[BW_11_O_39_]·13H_2_O	H_2_O		Reflux	20	90
7	ZnO	H_2_O		Reflux	30	91
8	Nd-B_2_W_22_/10	H_2_O		Reflux	20	90
9	Sm-B_2_W_22_/10	H_2_O		Reflux	15	95
10	Er-B_2_W_22_/10	H_2_O		Reflux	15	94
11	Ce-B_2_W_22_/10	H_2_O		Reflux	25	92
12	La-B_2_W_22_/10	H_2_O		Reflux	25	90
13	Gd-B_2_W_22_/10	H_2_O		Reflux	12	96
14	Gd-B_2_W_22_/15	H_2_O		Reflux	12	95
15	Gd-B_2_W_22_/10	H_2_O		r.t	25	80
16	Gd-B_2_W_22_/10	H_2_O		50 °C	20	91
17	Gd-B_2_W_22_/10	H_2_O/EtOH		Reflux	15	90
18	Gd-B_2_W_22_/10	EtOH		Reflux	20	92
19	Gd-B_2_W_22_/10	CH_2_Cl_2_		Reflux	25	90
20	Gd-B_2_W_22_/10	CH_3_CN		Reflux	25	85
21	Gd-B_2_W_22_/10	Tuloene		Reflux	30	85

The one-pot reaction of isatin, α-cyano compound (either malononitrile or ethylcyanoacetate), and 1,3-diketone (either ethyl acetoacetate, dimedone, or barbituric acid) or 3-methyl-1H-pyrazol-5(4H)-one/ 4-hydroxycoumarin or α-naphtol/β-naphtol) gave the favorite products. Notwithstanding, the effect of substituent on isatin ring, the yield of products was found in good to high. By employing acenaphthenequinone instead of isatin, the expected spiro-4H-pyrans were formed in good to high yields. The products obtained from ethylcyanoacetate need a longer reaction time than those obtained from malononitrile that possibly is due to the lower reactivity of ethylcyanoacetate (Table 3). All products were known and identified by comparing their melting points with authentic literature. Some selected NMR spectra are presented in supplementary file (Figs. S16–S47).

**Table 3:** Synthesis of spiro-2-amino-4H-pryans^37^.


Entry	Product	Time (min)	Yield %	TOF	M.p./ °C Obs	M.p. °C/Lit
1		12	96	320	295–298	298–299
2		15	96	256	251–254	253–255
3		8	94	482	290–293	290–292
4		10	92	368	255–258	256–258
5		16	92	230	299–302	> 300
6		20	91	182	253–254	251–253
7		22	90	163	241–243	240–242
8		25	90	144	261–263	262–264
9		20	91	182	270–272	273–275
10		25	90	144	208–211	207–209
11		20	94	188	236–239	236–237
12		22	90	163	232–235	235–236
13		22	91	165	286–287	285–286
14		21	92	175	280–282	280–281
15		22	89	164	239–242	242–243
16		16	91	233	245–248	245–247
17		10	95	395	269–273	268–270
18		14	92	262	257–260	259–262
19		18	93	206	> 300	> 300
20		24	88	146	195–198	194–196
21		26	91	141	> 300	> 300
22		21	90	171	> 300	> 300
23		25	91	144	256–258	258–260
24		22	94	174	195–198	193–196
25		25	95	151	297–299	298–299
26		24	93	155	248–249	247–248

**Scheme 1:**
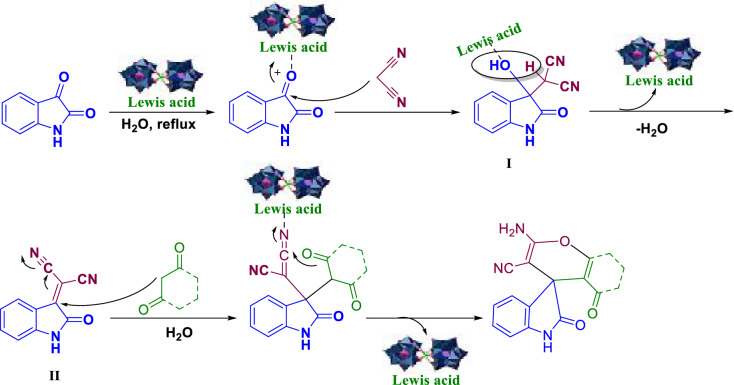
The reasonable mechanism for the synthesis of spirooxindole derivatives.

In Scheme 1, we propose a sensible mechanism for the preparation of spirooxindole derivatives. First, the **Gd-B**_**2**_**W**_**22**_ catalyst, as a Lewis acid, activates the carbonyl group of the isatin molecule, and then malononitrile, due to alpha-activated hydrogens, will have a nucleophilic attack on activated carbon, which produces intermediate 1. This intermediate creates intermediate 2 by elimination of water, and finally the corresponding product was synthesized by adding dimedone to this intermediate.

To confirm the wide effectiveness of nano-**Gd-B**_**2**_**W**_**22**_ as a catalyst, this was used in the reaction of isatin derivatives, 6-amino-1,3-dimethyl uracil, and 1,3-diketone (either dimedone, 1,3dimethyl barbituric acid, or barbituric acid). These reactions were successfully catalyzed by nano-**Gd-B**_**2**_**W**_**22**_ in refluxing water under optimized conditions furnishing spiro-products in good efficiency. The results are summarized in Table 4.

**Table 4:**
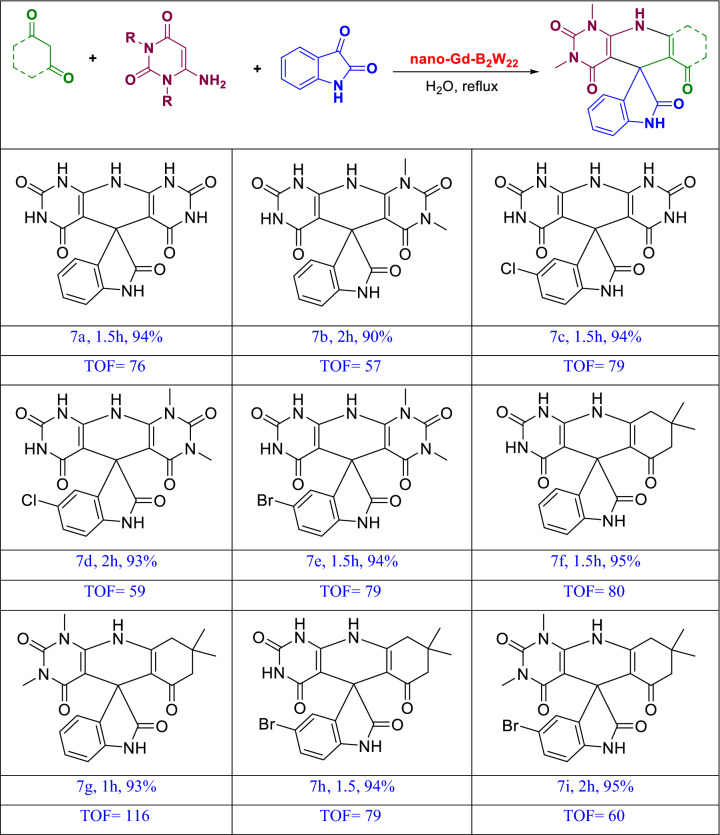
Synthesis of uracil-fused spirooxindoles^10,38,39^.

**Figure 10:**
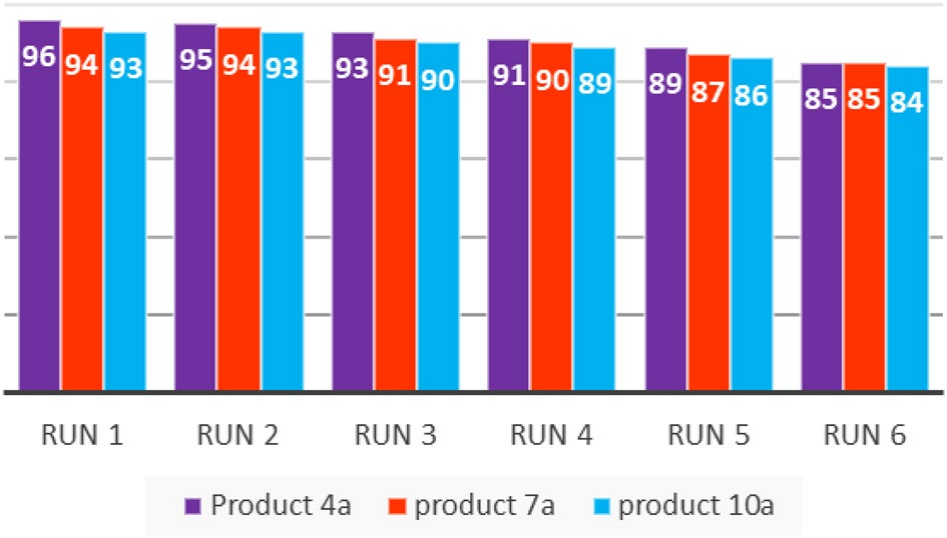
Reusability of nano-**Gd-B**_**2**_**W**_**22**_.

Next, the catalytic effect of nano-**Gd-B**_**2**_**W**_**22**_ was studied in the production of pyrroloacridine compounds through the one-pot reaction of isatin, aromatic amines, and dimedone. The generalization of this reaction was considered using different aromatic amine-bearing electron-donating and electron-withdrawing substituents. The expected pyrroloacridine derivatives were formed in wonderful yield within short reaction times as summarized in Table 5 (Fig. 10).

**Table 5:**
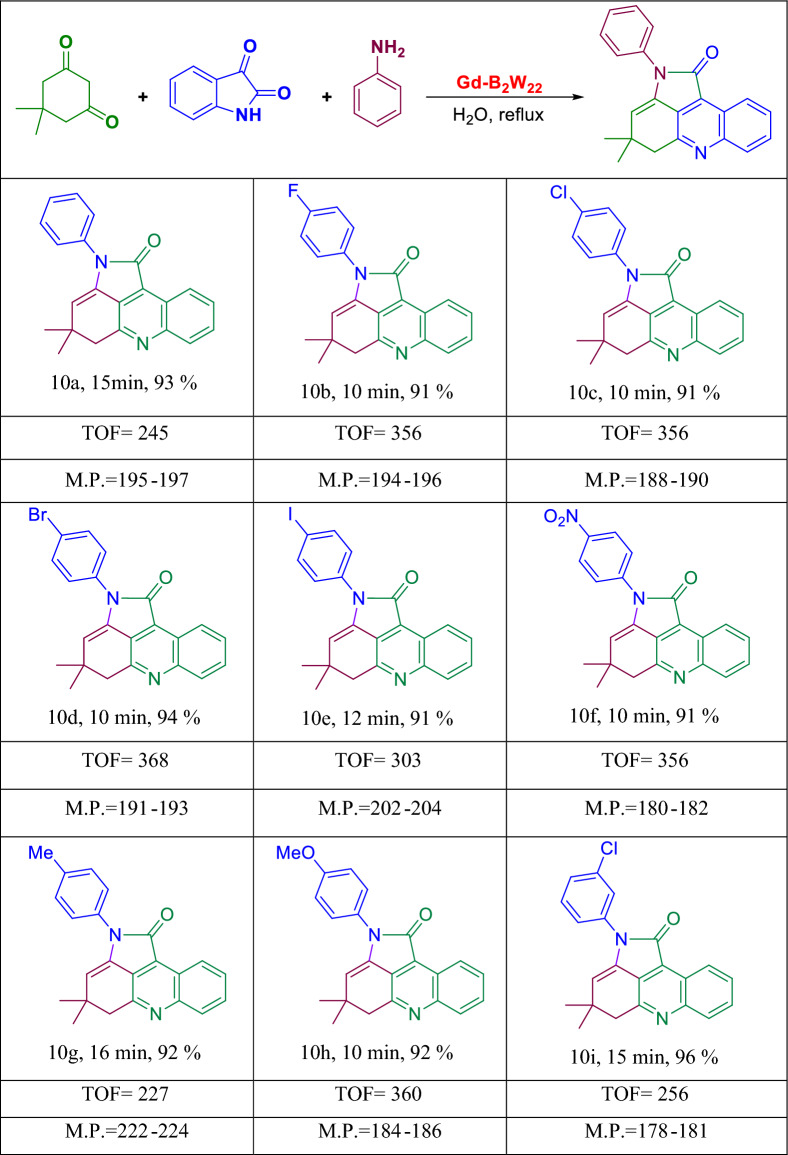
One-pot, three-component synthesis of pyrrolo[2,3,4-kl]acridin-1-one derivatives^45,46^.

### Catalyst recyclability

**Figure 11:**
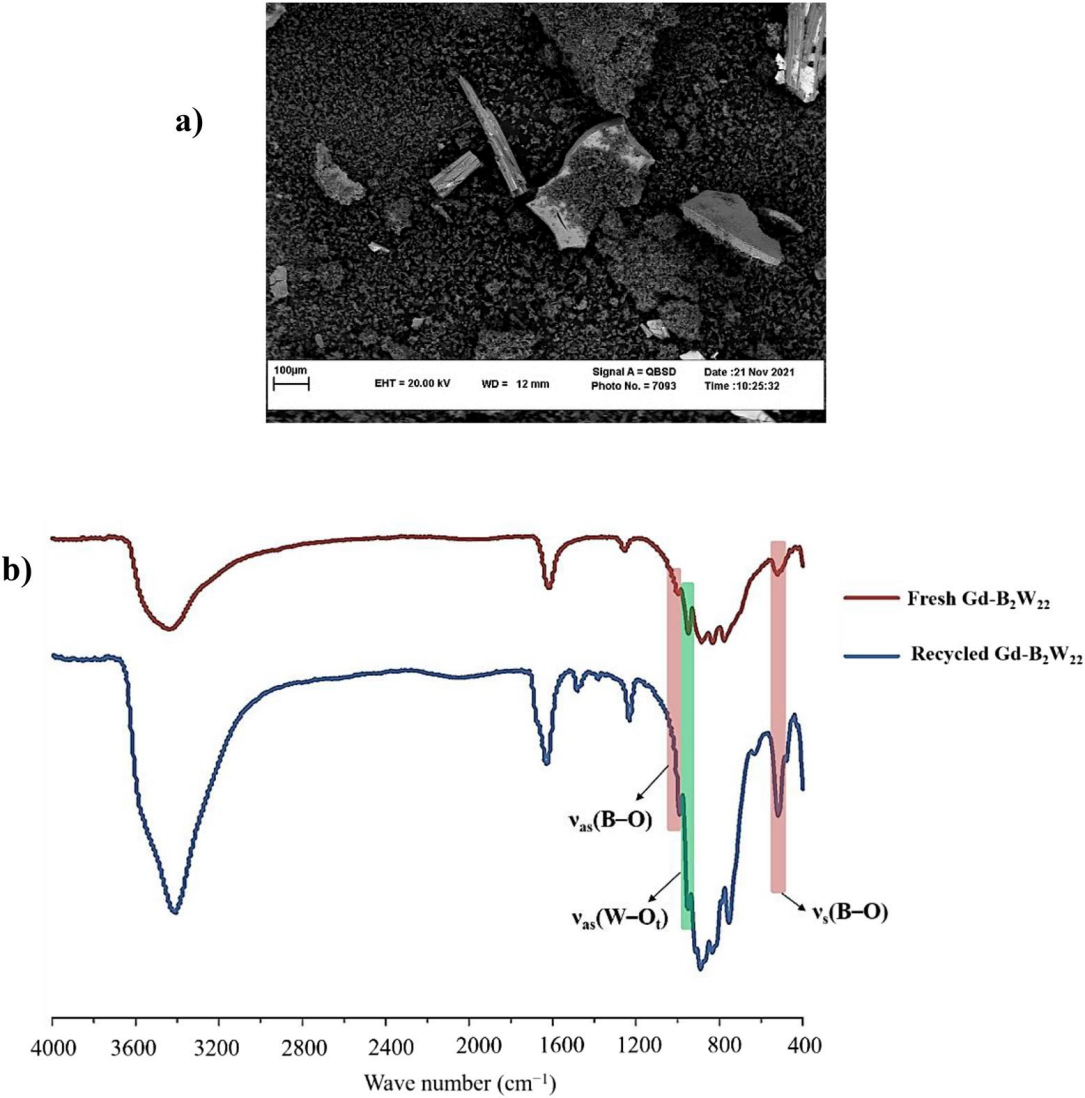
(**a**) SEM image of recycled nano-**Gd-B**_**2**_**W**_**22**_; (**b**) FTIR overlay of the fresh and recycled nano-**Gd-B**_**2**_**W**_**22**_.

Heterogeneous Catalysts, play an important and effective role in industries and other applications in laboratory scale. Hence, recyclability of the catalyst to prevent waste generation is one of the most important factors in catalysis. Nevertheless, recoverability of nano-**Gd-B**_**2**_**W**_**22**_ was evaluated on the model reaction and it was recycled up to 6 runs by simple filtration with a gradual decrease in activity from 96 to 85% in the corresponding product (Fig. 10). In addition, to elucidate whether the recycling process can result in any change in the catalyst’s morphology and structure, the SEM image as well as FTIR spectra of the recycled nano-**Gd-B**_**2**_**W**_**22**_ catalyst were recorded (Fig. 11). These results support that the structure of the nano-**Gd-B**_**2**_**W**_**22**_ underwent several reactions was preserved, but some agglomeration is evident.

## Concluding remarks

In the present study, a series of isostructural lanthanoid-containing polyoxometalate nanocatalysts **Ln-B**_**2**_**W**_**22**_ (Ln = La, Ce, Nd, Sm, Gd, and Er) were synthesized and characterized using a suite of analytical techniques. Among these nanocatalysts, the gadolinium-containing POM (**Gd-B**_**2**_**W**_**22**_) showed remarkable catalytic performance for the synthesis of bioactive isatin derivatives including spiro-2-amino-4H-pryans, uracil fused spirooxindoles, and pyrroloacridine derivatives under the reflux condition in high yields and short reaction times (8–26 min). Also, further studies are underway in our laboratory to extend the application of these family nanocatalysts to other coupling reactions.

## Supplementary Information

Supplementary Information.

## Data Availability

The raw/processed data that supports the findings of this study is available from the corresponding authors upon reasonable request.
