# Supplementary material for: Lanthanoid-containing polyoxometalate nanocatalysts in the synthesis of bioactive isatin-based compounds
Source: Sci Rep. 2022 Jul 14;12:12004. doi: 10.1038/s41598-022-16384-z (PMC9283471; doi:10.1038/s41598-022-16384-z)
Supplement: Supplementary file 1 — Supplementary Information. [file 41598_2022_16384_MOESM1_ESM.docx]

Supplementary Information Files for manuscript

**Lanthanoid-containing polyoxometalate** **nanocatalysts in the synthesis of bioactive** **isatin-based compounds**

Mansoureh Daraie ^a^, Masoud Mirzaei ^b,^*, Maryam Bazargan ^b^, Vadjiheh Sadat Amiri ^b#^, Bita Abdolahi Sanati ^b#^, Majid M. Heravi ^a,^**

^a^ Department of Chemistry, School of Sciences, Alzahra University, Vanak, Tehran, Iran.

^b^ Department of Chemistry, Faculty of Science, Ferdowsi University of Mashhad, Mashhad 9177948974, Iran.

^#^These authors contributed equally.

*Corresponding author: Masoud Mirzaei; E-mail: [mirzaeesh@um.ac.ir](mailto:mirzaeesh@um.ac.ir); ORCID number: 0000-0002-7256-4601

**Corresponding author: Majid M. Heravi; Email: [mmheravi@alzahra.ac.ir](mailto:mmheravi@alzahra.ac.ir); ORCID number: 0000-0002-9259-0591

Contents

[**Figure S1**. FTIR spectra of K_15_[Ln(BW_11_O_39_)_2_] (**Ln-B_2_W_22_**_;_ Ln = La, Ce, Nd, Sm, Gd and Er) and naked [BW_11_O_39_]^9−^ for comparison.. 2](#_Toc75021243)

[**Figure S2**. FTIR spectra of nano-**Ln-B_2_W_22_** (Ln = La, Ce, Nd, Sm, Gd and Er). 3](#_Toc75021244)

[**Figure S3.** Optical microscope images of the catalysts which reveal their crystal morphology..……………..4](#_Toc75021245)

[**Figure S4**. Particle size distribution histogram of nano-**La-B_2_W_22_**. 5](#_Toc75021245)

[**Figure S5**. Particle size distribution histogram of nano-**Ce-B_2_W_22_**.. 5](#_Toc75021245)

[**Figure S6**. Particle size distribution histogram of nano-**Nd-B_2_W_22_**.. 6](#_Toc75021245)

[**Figure S7**. Particle size distribution histogram of nano-**Sm-B_2_W_22_**.. 6](#_Toc75021245)

[**Figure S8**. Particle size distribution histogram of nano-**Er-B_2_W_22_**.. 7](#_Toc75021245)

[**Figure S9**. SEM images of nano-**La-B_2_W_22_**. 8](#_Toc75021246)

[**Figure S10**. EDS spectrum of nano-**La-B_2_W_22_**. 9](#_Toc75021247)

[**Figure S11**. EDS spectrum of nano-**Ce-B_2_W_22_**. 9](#_Toc75021247)

[**Figure S12**. EDS spectrum of nano-**Nd-B_2_W_22_**. 10](#_Toc75021247)

[**Figure S13**. EDS spectrum of nano-**Sm-B_2_W_22_**. 10](#_Toc75021247)

**[Figure S14](#_Toc75021247)**[. EDS spectrum of nano-](#_Toc75021247)**[Er-B](#_Toc75021247)_[2](#_Toc75021247)_[W](#_Toc75021247)_[22](#_Toc75021247)_**[. 11](#_Toc75021247)

**[Figure S15](#_Toc75021247)**[. Powder XRD patterns of nano-](#_Toc75021247)**[Ln-B](#_Toc75021247)_[2](#_Toc75021247)_[W](#_Toc75021247)_[22](#_Toc75021247)_** [(Ln = La, Ce, Nd, Sm, Gd and Er). 11](#_Toc75021247)

**[Figure S16](#_Toc75021247)**[.](#_Toc75021247) ^[1](#_Toc75021247)^[H NMR spectrum of 6-amino-5-cyano-3,4-dimethyl-N-phenyl-1,4-dihydropyrano[2,3-c]pyrazole-4-carboxamide. 12](#_Toc75021247)

[**Figure S17**. ^13^C NMR spectrum of 6-amino-5-cyano-3,4-dimethyl-N-phenyl-1,4-dihydropyrano[2,3-c]pyrazole-4-carboxamide. 12](#_Toc75021247)

[**Figure S18**. FTIR spectrum of 6-amino-5-cyano-3,4-dimethyl-N,1-diphenyl-1,4-dihydropyrano[2,3-c]pyrazole-4-carboxamide. 13](#_Toc75021247)

[**Figure S19**. ^1^H NMR spectrum of 6-amino-5-cyano-3,4-dimethyl-N,1-diphenyl-1,4-dihydropyrano[2,3-c]pyrazole-4-carboxamide. 13](#_Toc75021247)

[**Figure S20.** Mass spectrum of 2-amino-4-formyl-7,7-dimethyl-4-(8-methylnaphthalen-1-yl)-5-oxo-5,6,7,8-tetrahydro-4H-chromene-3-carbonitrile 14](#_Toc75021247)

[**Figure S21.** FTIR spectrum of 2-amino-4-formyl-7,7-dimethyl-4-(8-methylnaphthalen-1-yl)-5-oxo-5,6,7,8-tetrahydro-4H-chromene-3-carbonitrile. 14](#_Toc75021247)

[**Figure S22**. ^1^H NMR spectrum of 2-amino-4-formyl-7,7-dimethyl-4-(8-methylnaphthalen-1-yl)-5-oxo-5,6,7,8-tetrahydro-4H-chromene-3-carbonitrile. 15](#_Toc75021247)

[**Figure S23**. ^13^C NMR spectrum of 2-amino-4-formyl-7,7-dimethyl-4-(8-methylnaphthalen-1-yl)-5-oxo-5,6,7,8-tetrahydro-4H-chromene-3-carbonitrile. 15](#_Toc75021247)

[**Figure S24**. Mass spectrum of ethyl 2-amino-4-formyl-7,7-dimethyl-4-(8-methylnaphthalen-1-yl)-5-oxo-5,6,7,8-tetrahydro-4H-chromene-3-carboxylate. 16](#_Toc75021247)

[**Figure S25**. FTIR spectrum of ethyl 2-amino-4-formyl-7,7-dimethyl-4-(8-methylnaphthalen-1-yl)-5-oxo-5,6,7,8-tetrahydro-4H-chromene-3-carboxylate. 16](#_Toc75021247)

[**Figure S26**. ^1^H NMR spectrum of ethyl 2-amino-4-formyl-7,7-dimethyl-4-(8-methylnaphthalen-1-yl)-5-oxo-5,6,7,8-tetrahydro-4H-chromene-3-carboxylate. 17](#_Toc75021247)

[**Figure S27**. ^13^C NMR spectrum of ethyl 2-amino-4-formyl-7,7-dimethyl-4-(8-methylnaphthalen-1-yl)-5-oxo-5,6,7,8-tetrahydro-4H-chromene-3-carboxylate. 17](#_Toc75021247)

[**Figure S28**. Mass spectrum of 7-amino-5-formyl-5-(8-methylnaphthalen-1-yl)-2,4-dioxo-1,3,4,5-tetrahydro-2H-pyrano[2,3-d]pyrimidine-6-carbonitrile. 18](#_Toc75021247)

[**Figure S29**. FTIR spectrum of 7-amino-5-formyl-5-(8-methylnaphthalen-1-yl)-2,4-dioxo-1,3,4,5-tetrahydro-2H-pyrano[2,3-d]pyrimidine-6-carbonitrile. 18](#_Toc75021247)

[**Figure S30**. ^1^H NMR spectrum of 7-amino-5-formyl-5-(8-methylnaphthalen-1-yl)-2,4-dioxo-1,3,4,5-tetrahydro-2H-pyrano[2,3-d]pyrimidine-6-carbonitrile. 19](#_Toc75021247)

[**Figure S31**. ^13^C NMR spectrum of 7-amino-5-formyl-5-(8-methylnaphthalen-1-yl)-2,4-dioxo-1,3,4,5-tetrahydro-2H-pyrano[2,3-d]pyrimidine-6-carbonitrile. 19](#_Toc75021247)

[**Figure S32**. Mass spectrum of 3-amino-1-formyl-1-(8-methylnaphthalen-1-yl)-1H-benzo[f]chromene-2-carbonitrile. 20](#_Toc75021247)

[**Figure S33**. FTIR spectrum of 3-amino-1-formyl-1-(8-methylnaphthalen-1-yl)-1H-benzo[f]chromene-2-carbonitrile. 20](#_Toc75021247)

[**Figure S34**. ^1^H NMR spectrum of 3-amino-1-formyl-1-(8-methylnaphthalen-1-yl)-1H-benzo[f]chromene-2-carbonitrile. 21](#_Toc75021247)

[**Figure S35**. ^13^C NMR spectrum of 3-amino-1-formyl-1-(8-methylnaphthalen-1-yl)-1H-benzo[f]chromene-2-carbonitrile. 21](#_Toc75021247)

[**Figure S36**. Mass spectrum of 2-amino-4-formyl-4-(8-methylnaphthalen-1-yl)-5-oxo-4H,5H-pyrano[3,2-c]chromene-3-carbonitrile 22](#_Toc75021247)

[**Figure S37**. FTIR spectrum of 2-amino-4-formyl-4-(8-methylnaphthalen-1-yl)-5-oxo-4H,5H-pyrano[3,2-c]chromene-3-carbonitrile 22](#_Toc75021247)

[**Figure S38**. ^1^H NMR spectrum of 2-amino-4-formyl-4-(8-methylnaphthalen-1-yl)-5-oxo-4H,5H-pyrano[3,2-c]chromene-3-carbonitrile. 23](#_Toc75021247)

[**Figure S39**. ^13^C NMR spectrum of 2-amino-4-formyl-4-(8-methylnaphthalen-1-yl)-5-oxo-4H,5H-pyrano[3,2-c]chromene-3-carbonitrile. 23](#_Toc75021247)

[**Figure S40**. Mass spectrum of 6-amino-4-formyl-3-methyl-4-(8-methylnaphthalen-1-yl)-1,4-dihydropyrano[2,3-c]pyrazole-5-carbonitrile 24](#_Toc75021247)

[**Figure S41**. FTIR spectrum of 6-amino-4-formyl-3-methyl-4-(8-methylnaphthalen-1-yl)-1,4-dihydropyrano[2,3-c]pyrazole-5-carbonitrile. 24](#_Toc75021247)

[**Figure S42**. ^1^H NMR spectrum of 6-amino-4-formyl-3-methyl-4-(8-methylnaphthalen-1-yl)-1,4-dihydropyrano[2,3-c]pyrazole-5-carbonitrile. 25](#_Toc75021247)

[**Figure S43**. ^13^C NMR spectrum of 6-amino-4-formyl-3-methyl-4-(8-methylnaphthalen-1-yl)-1,4-dihydropyrano[2,3-c]pyrazole-5-carbonitrile. 25](#_Toc75021247)

[**Figure S44**. Mass spectrum of ethyl 6-amino-4-formyl-3-methyl-4-(8-methylnaphthalen-1-yl)-1,4-dihydropyrano[2,3-c]pyrazole-5-carboxylate. 26](#_Toc75021247)

[**Figure S45**. FTIR spectrum of ethyl 6-amino-4-formyl-3-methyl-4-(8-methylnaphthalen-1-yl)-1,4-dihydropyrano[2,3-c]pyrazole-5-carboxylate. 26](#_Toc75021247)

[**Figure S46**. ^1^H NMR spectrum of ethyl 6-amino-4-formyl-3-methyl-4-(8-methylnaphthalen-1-yl)-1,4-dihydropyrano[2,3-c]pyrazole-5-carboxylate. 27](#_Toc75021247)

[**Figure S47**. ^13^C NMR spectrum of ethyl 6-amino-4-formyl-3-methyl-4-(8-methylnaphthalen-1-yl)-1,4-dihydropyrano[2,3-c]pyrazole-5-carboxylate.. 27](#_Toc75021247)


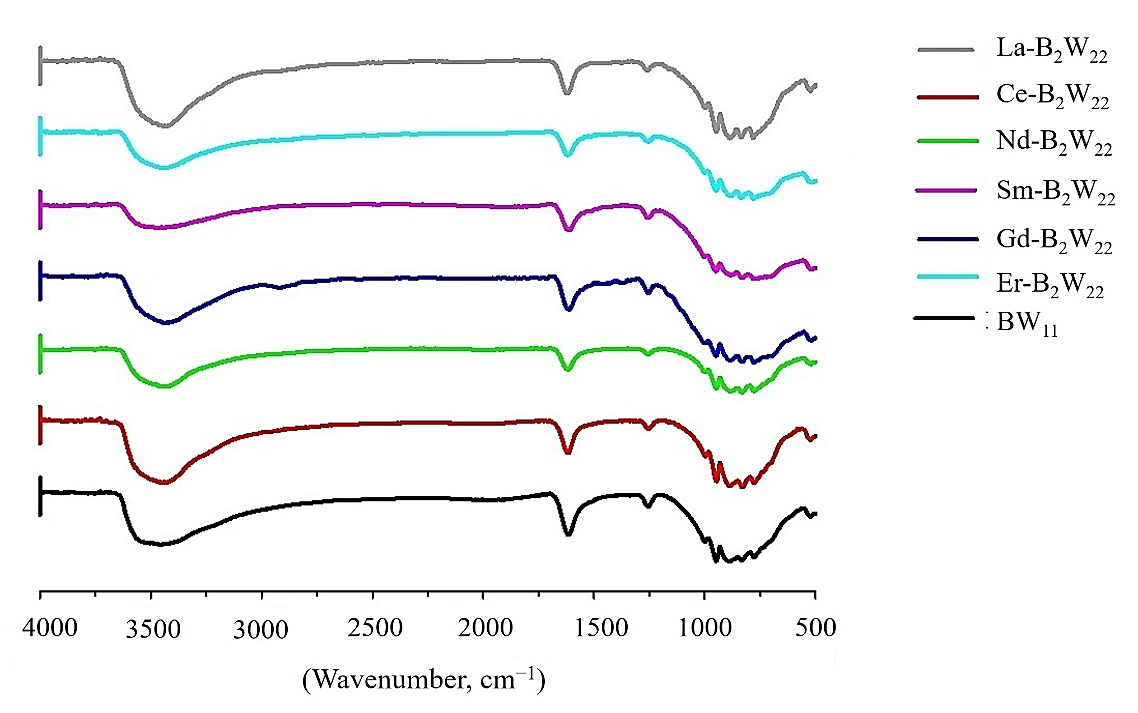


**Figure S1.** FTIR spectra of K_15_[Ln(BW_11_O_39_)_2_] (**Ln-B_2_W_22_**_;_ Ln = La, Ce, Nd, Sm, Gd and Er) and naked [BW_11_O_39_]^9−^ for comparison.


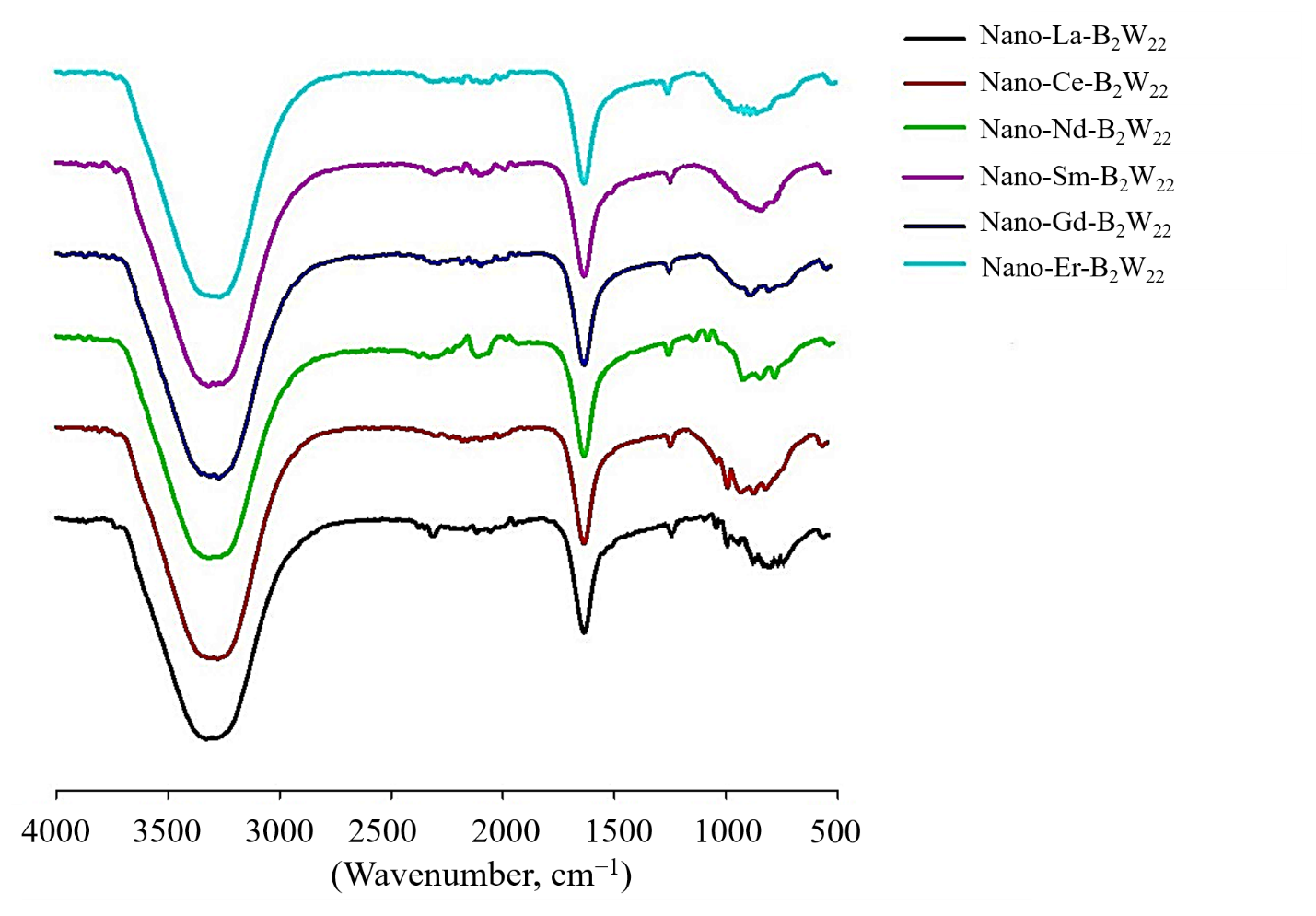


**Figure S2.** FTIR spectra of nano-**Ln-B_2_W_22_** (Ln = La, Ce, Nd, Sm, Gd and Er).


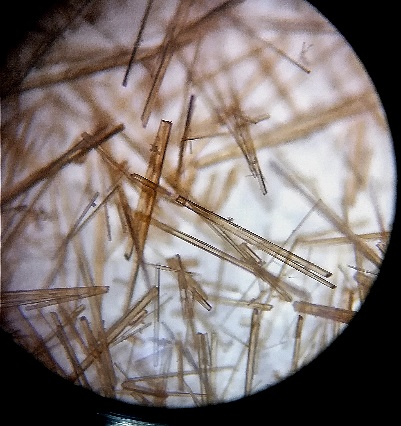


**Ce-B_2_W_22_**

**Nd-B_2_W_22_**


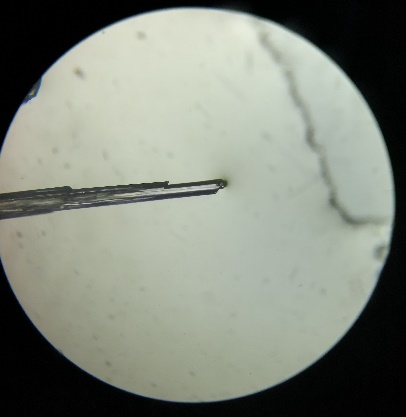


**La-B_2_W_22_**


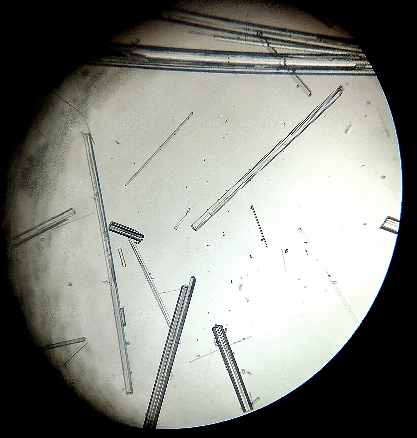

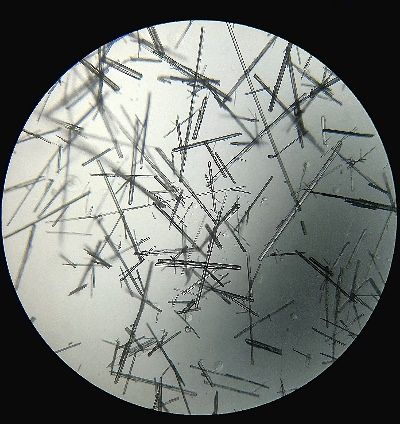


**Er-B_2_W_22_**


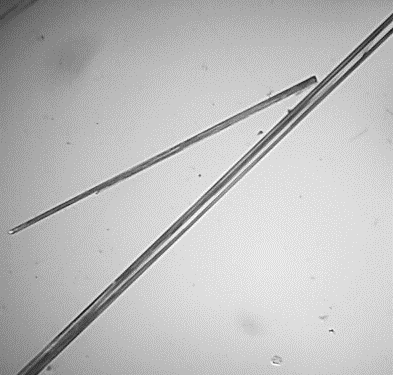


**Gd-B_2_W_22_**


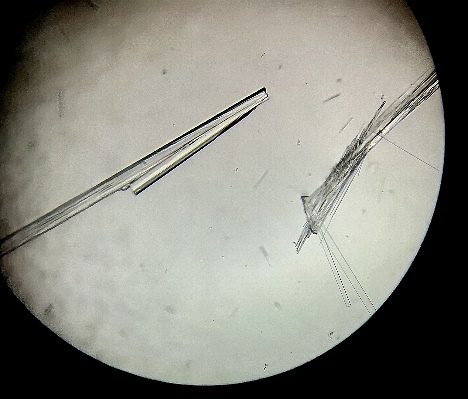


**Sm-B_2_W_22_**

**Figure S3.** Optical microscope images of the catalysts which reveal their crystal morphology.


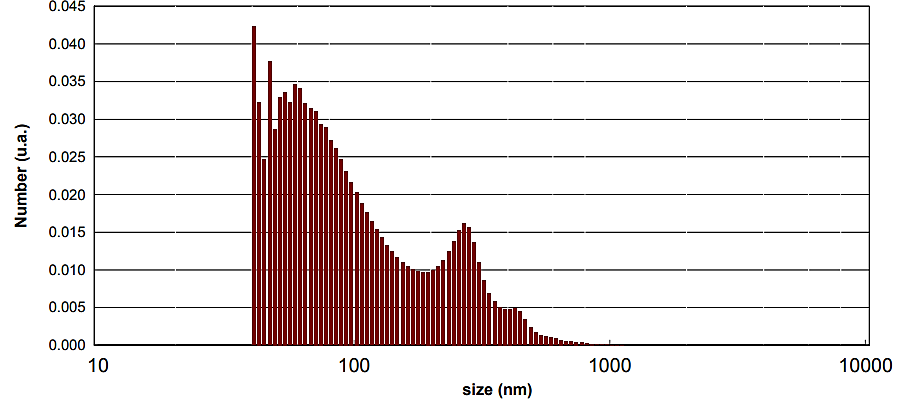


**Figure S4.** Particle size distribution histogram of nano-**La-B_2_W_22_**.


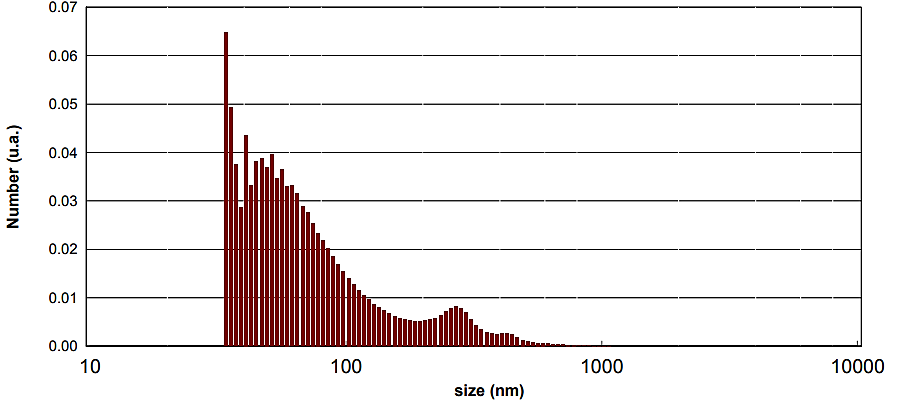


**Figure S5.** Particle size distribution histogram of nano-**Ce-B_2_W_22_**.


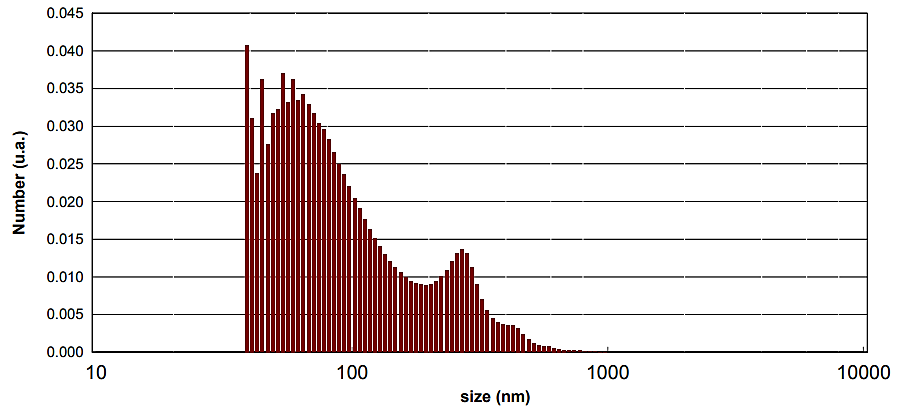


**Figure S6.** Particle size distribution histogram of nano-**Nd-B_2_W_22_**.


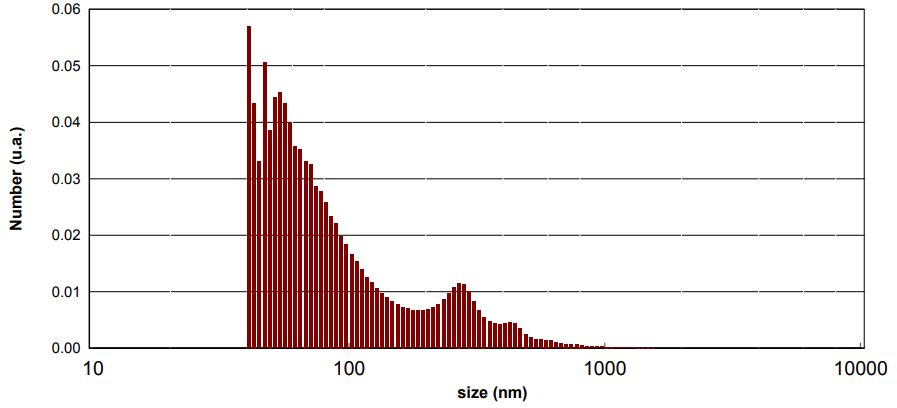


**Figure S7.** Particle size distribution histogram of nano-**Sm-B_2_W_22_**.


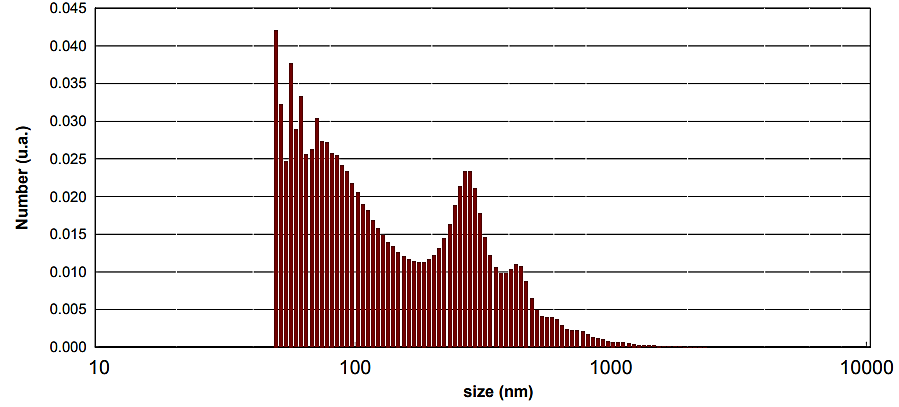


**Figure S8.** Particle size distribution histogram of nano-**Er-B_2_W_22_**.


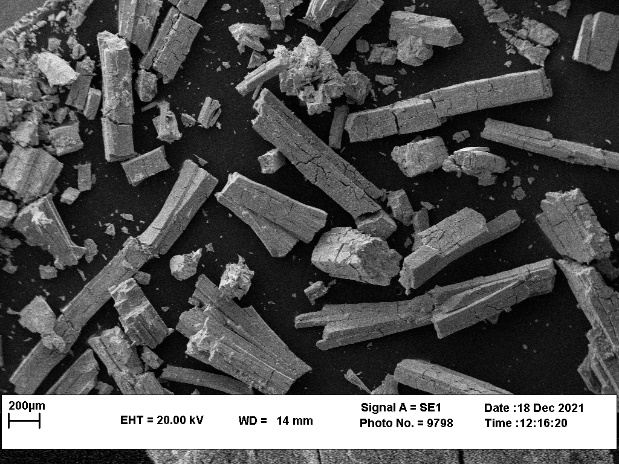

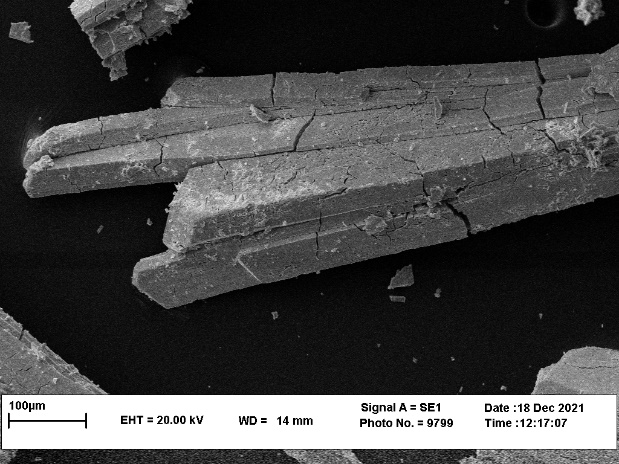


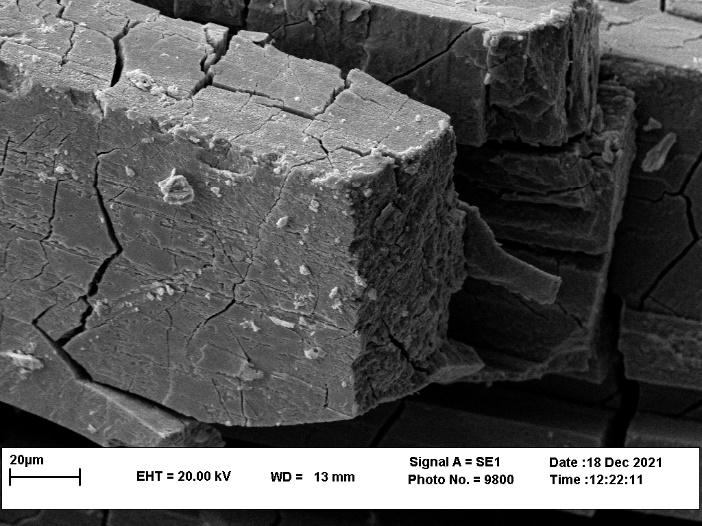


**Figure S9**. SEM images of **La-B_2_W_22_**_._


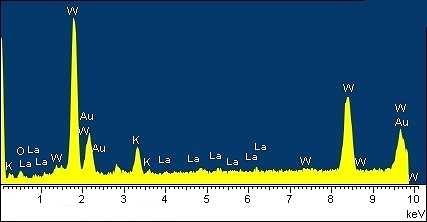


| Element | Weight% |
| --- | --- |
| O | 19.61 |
| K | 9.31 |
| La | 2.82 |
| W | 68.26 |
| Totals | 100.00 |

**Figure S10.** EDS spectrum of nano-**La-B_2_W_22_**.


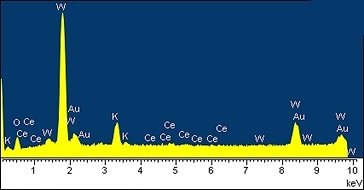


| Element | Weight% |
| --- | --- |
| O | 16.85 |
| K | 8.57 |
| Ce | 3.56 |
| W | 71.02 |
| Totals | 100.00 |

**Figure S11.** EDS spectrum of nano-**Ce-B_2_W_22_**.


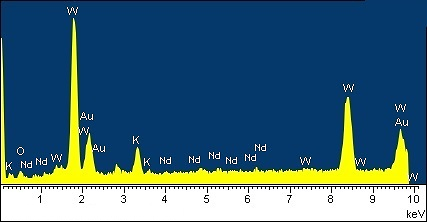


| Element | Weight% |
| --- | --- |
| O | 22.40 |
| K | 9.01 |
| Nd | 3.12 |
| W | 65.47 |
| Totals | 100.00 |

**Figure S12.** EDS spectrum of nano-**Nd-B_2_W_22_**.


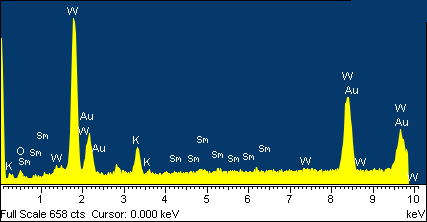


| Element | Weight% |
| --- | --- |
| O | 21.14 |
| K | 7.98 |
| Sm | 2.78 |
| W | 68.10 |
| Totals | 100.00 |

**Figure S13.** EDS spectrum of nano-**Sm-B_2_W_22_**.


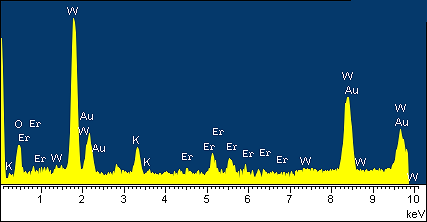


| Element | Weight% |
| --- | --- |
| O | 20.93 |
| K | 9.31 |
| Er | 2.52 |
| W | 67.24 |
| Totals | 100.00 |

**Figure S14.** EDS spectrum of nano-**Er-B_2_W_22_**.


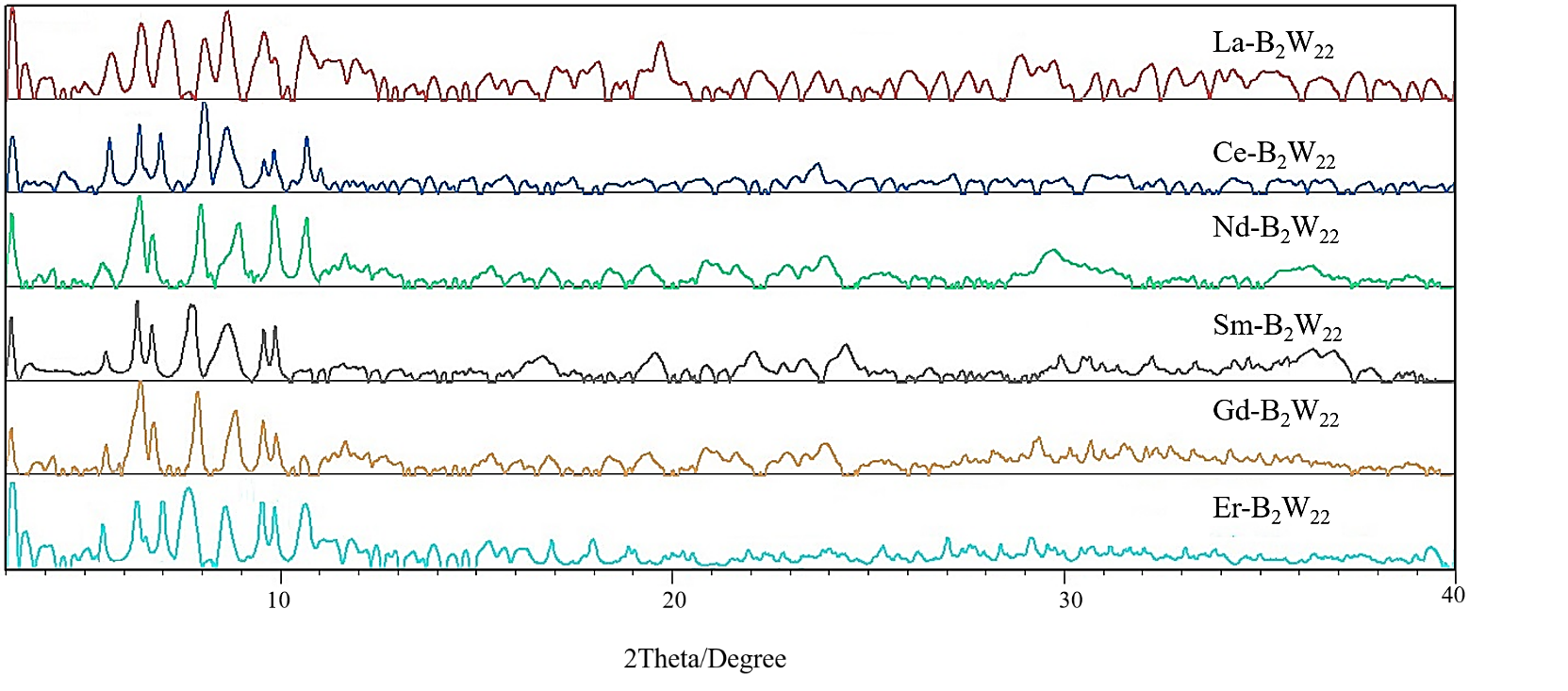


**Figure S15**. Powder XRD patterns of nano-**Ln-B_2_W_22_** (Ln = La, Ce, Nd, Sm, Gd and Er).


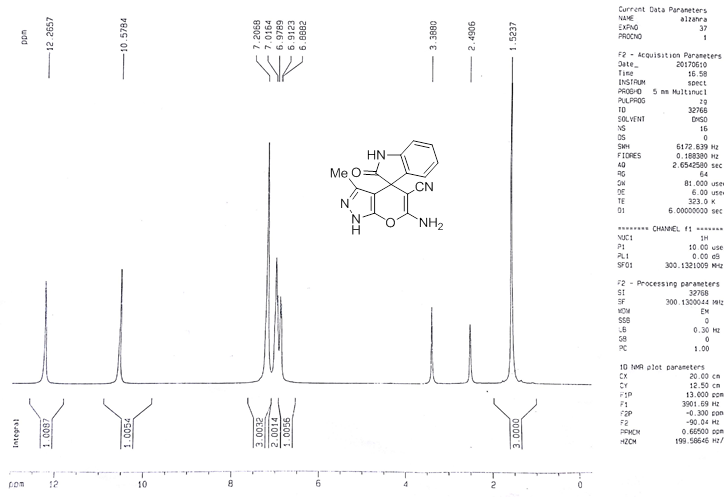


**Figure S16.**  ^1^H NMR spectrum of 6-amino-5-cyano-3,4-dimethyl-N-phenyl-1,4-dihydropyrano[2,3-c]pyrazole-4-carboxamide.


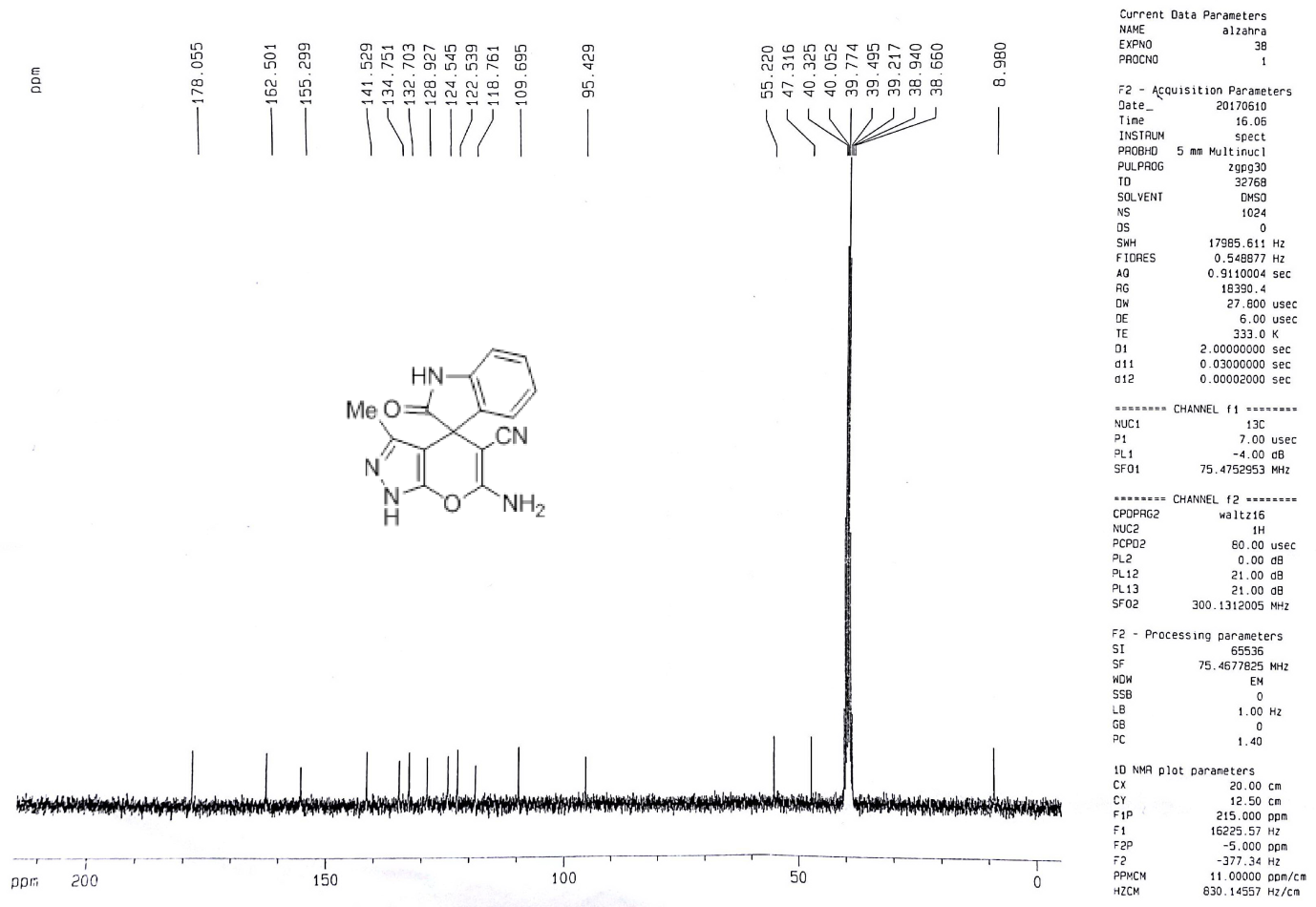
**Figure S17.** ^13^C NMR spectrum of 6-amino-5-cyano-3,4-dimethyl-N-phenyl-1,4-dihydropyrano[2,3-c]pyrazole-4-carboxamide.


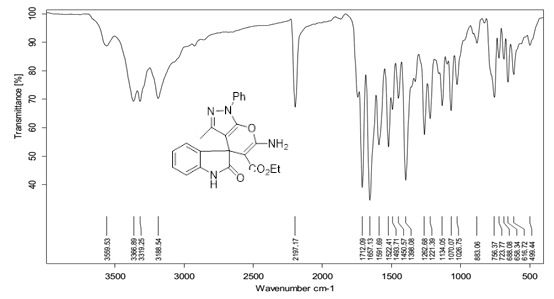


**Figure S18.** FTIR spectrum of 6-amino-5-cyano-3,4-dimethyl-N,1-diphenyl-1,4-dihydropyrano[2,3-c]pyrazole-4-carboxamide.


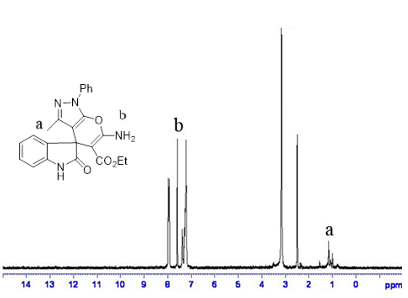


**Figure S19.** ^1^H NMR spectrum of 6-amino-5-cyano-3,4-dimethyl-N,1-diphenyl-1,4-dihydropyrano[2,3-c]pyrazole-4-carboxamide.


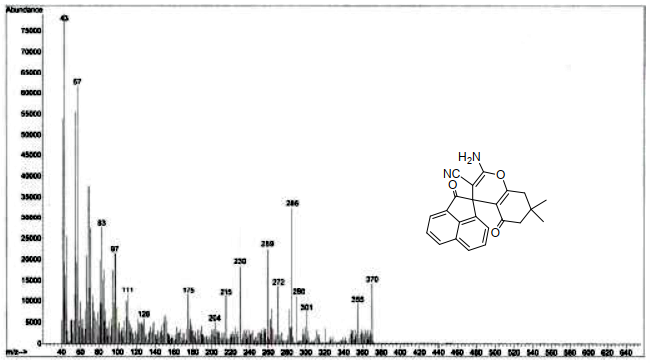


**Figure S20.** Mass spectrum of 2-amino-4-formyl-7,7-dimethyl-4-(8-methylnaphthalen-1-yl)-5-oxo-5,6,7,8-tetrahydro-4H-chromene-3-carbonitrile.


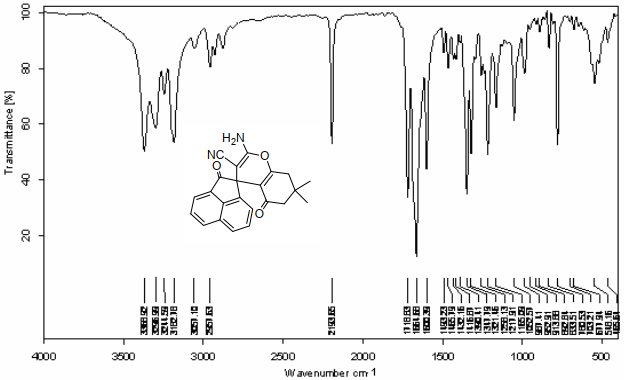


**Figure S21.** FTIR spectrum of 2-amino-4-formyl-7,7-dimethyl-4-(8-methylnaphthalen-1-yl)-5-oxo-5,6,7,8-tetrahydro-4H-chromene-3-carbonitrile.


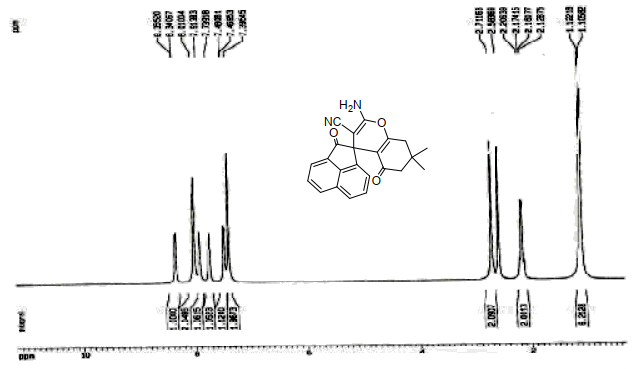


**Figure S22.** ^1^H NMR spectrum of 2-amino-4-formyl-7,7-dimethyl-4-(8-methylnaphthalen-1-yl)-5-oxo-5,6,7,8-tetrahydro-4H-chromene-3-carbonitrile.


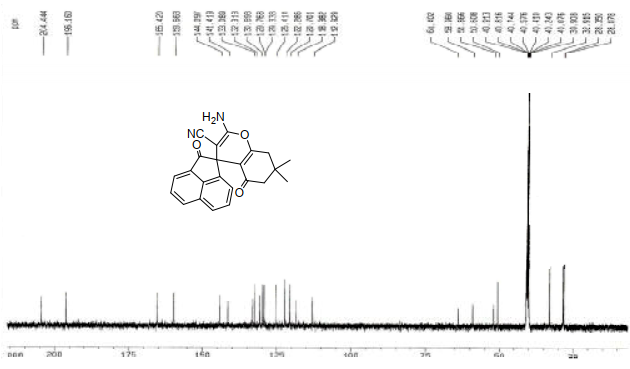


**Figure S23.** ^13^C NMR spectrum of 2-amino-4-formyl-7,7-dimethyl-4-(8-methylnaphthalen-1-yl)-5-oxo-5,6,7,8-tetrahydro-4H-chromene-3-carbonitrile.


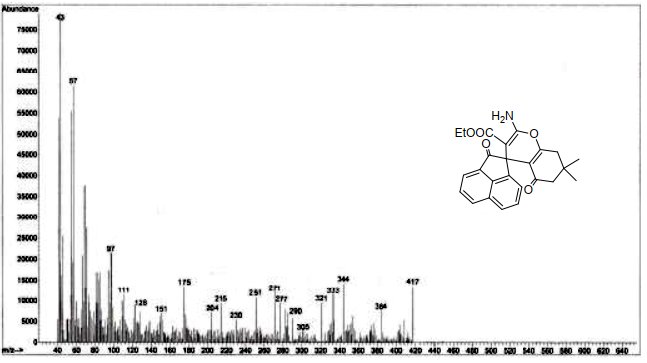


**Figure S24.** Mass spectrum of ethyl 2-amino-4-formyl-7,7-dimethyl-4-(8-methylnaphthalen-1-yl)-5-oxo-5,6,7,8-tetrahydro-4H-chromene-3-carboxylate.


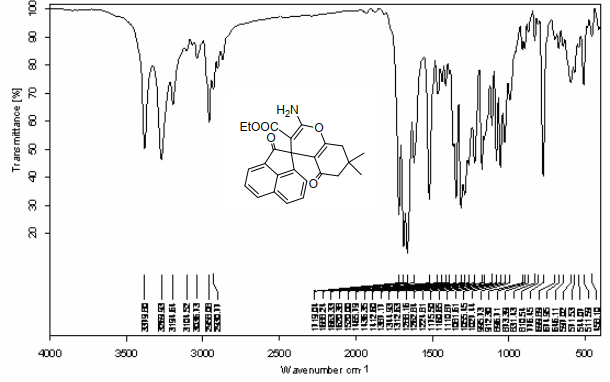


**Figure S25.** FTIR spectrum of ethyl 2-amino-4-formyl-7,7-dimethyl-4-(8-methylnaphthalen-1-yl)-5-oxo-5,6,7,8-tetrahydro-4H-chromene-3-carboxylate.


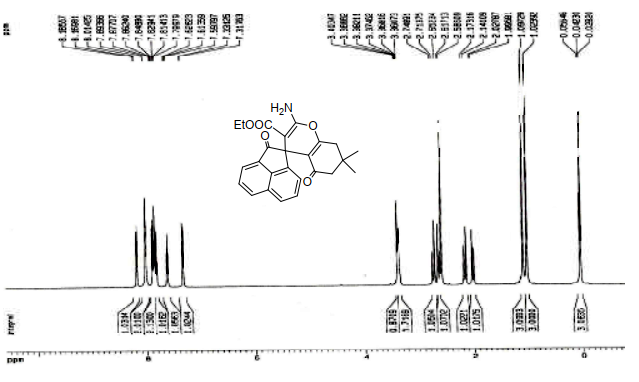


**Figure S26.** ^1^H NMR spectrum of ethyl 2-amino-4-formyl-7,7-dimethyl-4-(8-methylnaphthalen-1-yl)-5-oxo-5,6,7,8-tetrahydro-4H-chromene-3-carboxylate.


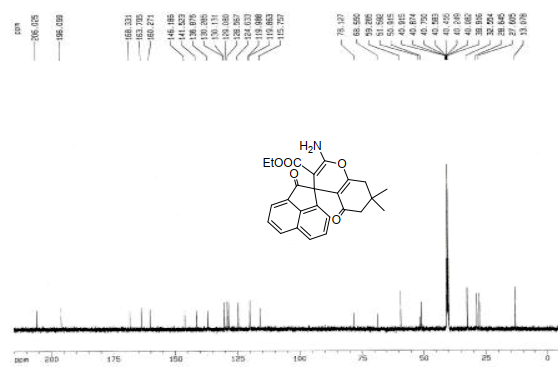


**Figure S27.** ^13^C NMR spectrum of ethyl 2-amino-4-formyl-7,7-dimethyl-4-(8-methylnaphthalen-1-yl)-5-oxo-5,6,7,8-tetrahydro-4H-chromene-3-carboxylate.


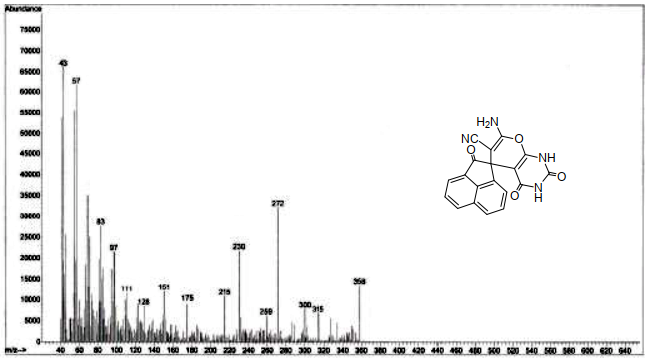


**Figure S28.** Mass spectrum of 7-amino-5-formyl-5-(8-methylnaphthalen-1-yl)-2,4-dioxo-1,3,4,5-tetrahydro-2H-pyrano[2,3-d]pyrimidine-6-carbonitrile.


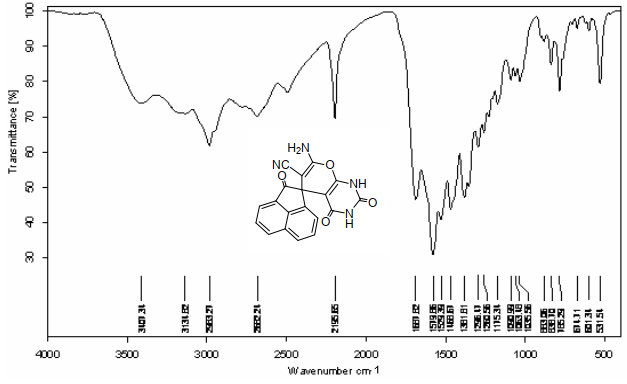


**Figure S29.** FTIR spectrum of 7-amino-5-formyl-5-(8-methylnaphthalen-1-yl)-2,4-dioxo-1,3,4,5-tetrahydro-2H-pyrano[2,3-d]pyrimidine-6-carbonitrile.


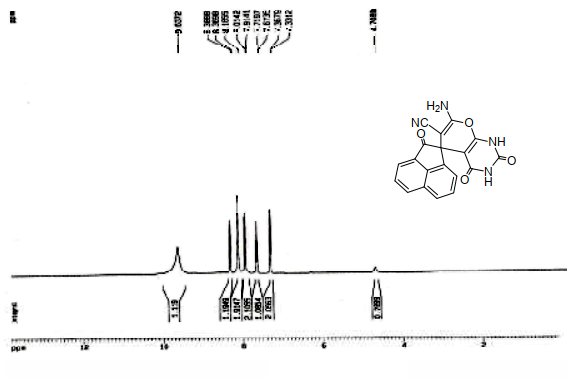


**Figure S30.** ^1^H NMR spectrum of 7-amino-5-formyl-5-(8-methylnaphthalen-1-yl)-2,4-dioxo-1,3,4,5-tetrahydro-2H-pyrano[2,3-d]pyrimidine-6-carbonitrile.


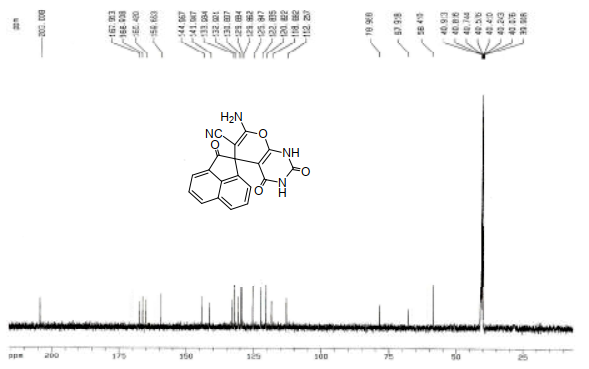


**Figure S31.** ^13^C NMR spectrum of 7-amino-5-formyl-5-(8-methylnaphthalen-1-yl)-2,4-dioxo-1,3,4,5-tetrahydro-2H-pyrano[2,3-d]pyrimidine-6-carbonitrile.


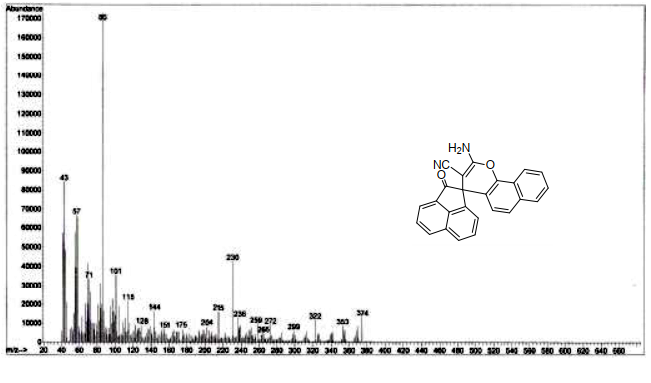


**Figure S32.** Mass spectrum of 3-amino-1-formyl-1-(8-methylnaphthalen-1-yl)-1H-benzo[f]chromene-2-carbonitrile.


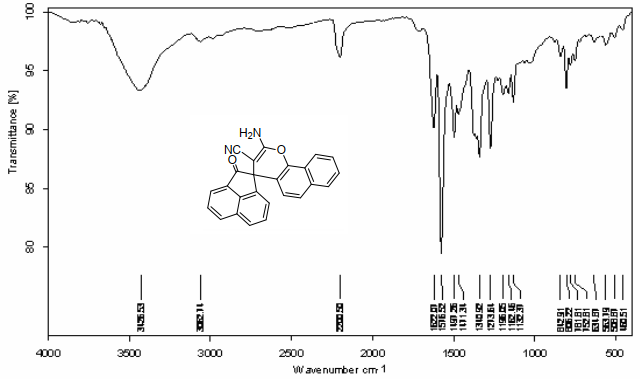


**Figure S33.** FTIR spectrum of 3-amino-1-formyl-1-(8-methylnaphthalen-1-yl)-1H-benzo[f]chromene-2-carbonitrile.


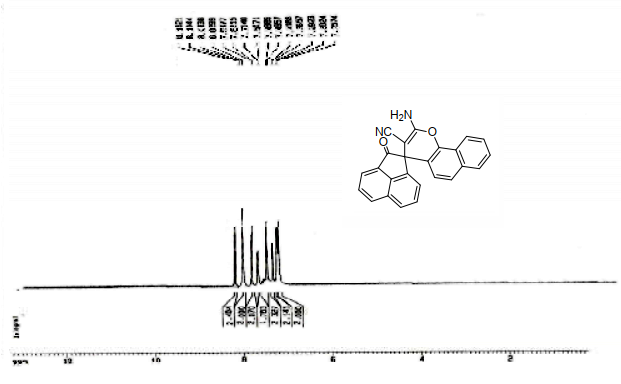


**Figure S34.** ^1^H NMR spectrum of 3-amino-1-formyl-1-(8-methylnaphthalen-1-yl)-1H-benzo[f]chromene-2-carbonitrile.


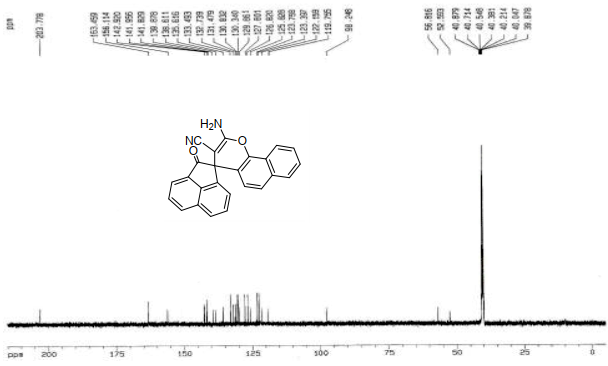


**Figure S35.** ^13^C NMR spectrum of 3-amino-1-formyl-1-(8-methylnaphthalen-1-yl)-1H-benzo[f]chromene-2-carbonitrile.


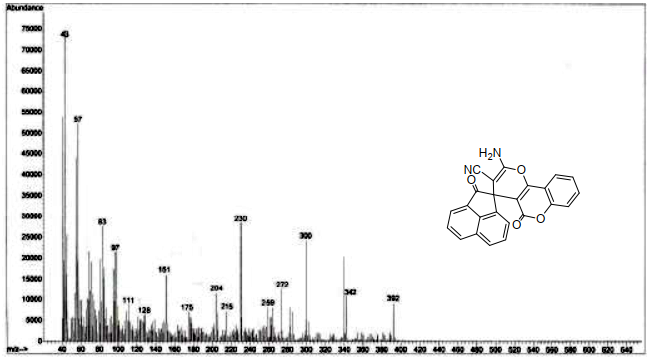


**Figure S36.** Mass spectrum of 2-amino-4-formyl-4-(8-methylnaphthalen-1-yl)-5-oxo-4H,5H-pyrano[3,2-c]chromene-3-carbonitrile.


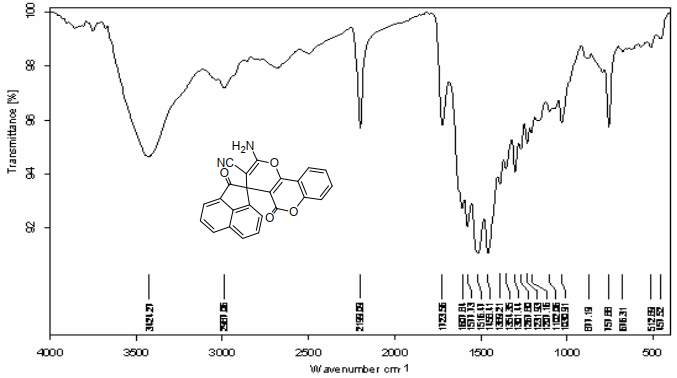


**Figure S37.** FTIR spectrum of 2-amino-4-formyl-4-(8-methylnaphthalen-1-yl)-5-oxo-4H,5H-pyrano[3,2-c]chromene-3-carbonitrile.


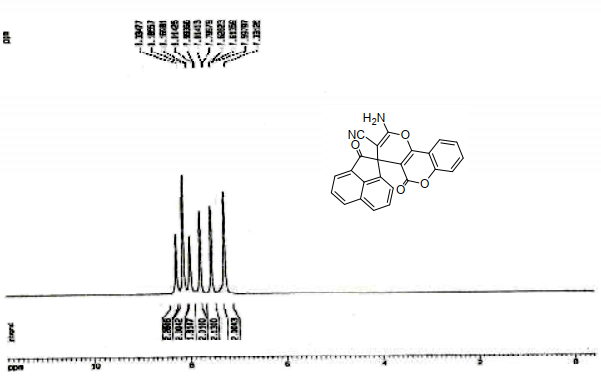


**Figure S38.** ^1^H NMR spectrum of 2-amino-4-formyl-4-(8-methylnaphthalen-1-yl)-5-oxo-4H,5H-pyrano[3,2-c]chromene-3-carbonitrile.


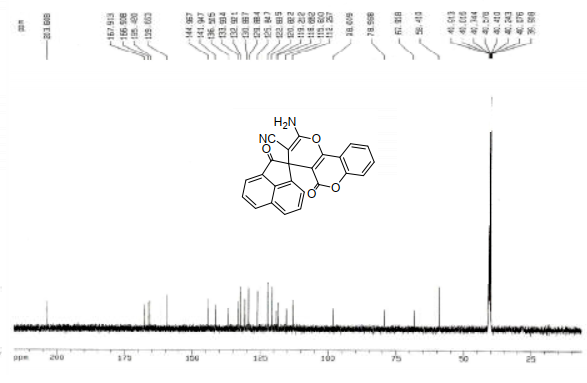


**Figure S39.** ^13^C NMR spectrum of 2-amino-4-formyl-4-(8-methylnaphthalen-1-yl)-5-oxo-4H,5H-pyrano[3,2-c]chromene-3-carbonitrile.


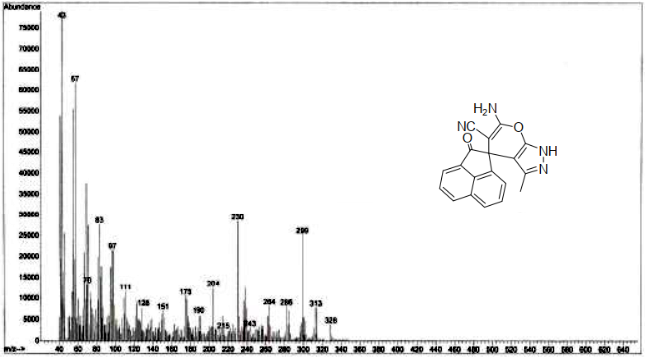


**Figure S40.** Mass spectrum of 6-amino-4-formyl-3-methyl-4-(8-methylnaphthalen-1-yl)-1,4-dihydropyrano[2,3-c]pyrazole-5-carbonitrile.


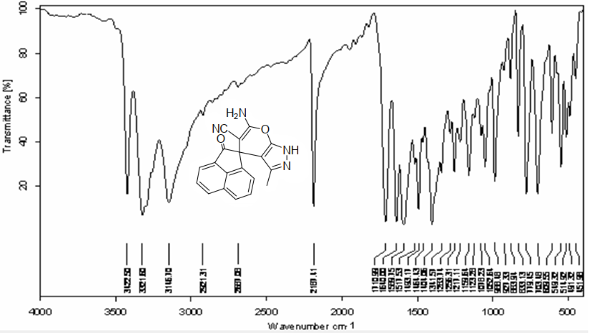


**Figure S41.** FTIR spectrum of 6-amino-4-formyl-3-methyl-4-(8-methylnaphthalen-1-yl)-1,4-dihydropyrano[2,3-c]pyrazole-5-carbonitrile.


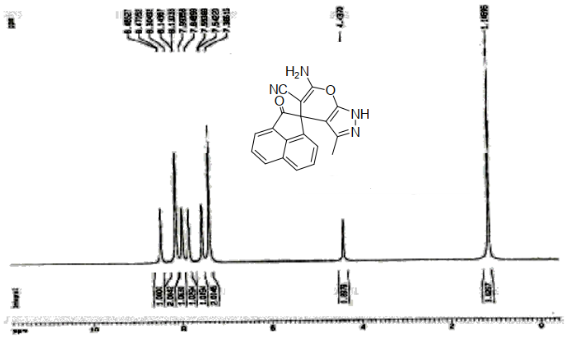


**Figure S42.** ^1^H NMR spectrum of 6-amino-4-formyl-3-methyl-4-(8-methylnaphthalen-1-yl)-1,4-dihydropyrano[2,3-c]pyrazole-5-carbonitrile.


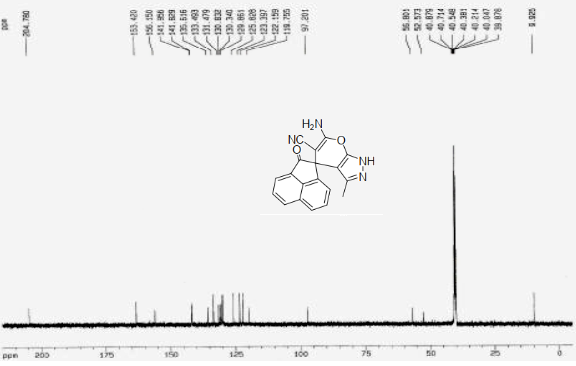


**Figure S43.** ^13^C NMR spectrum of 6-amino-4-formyl-3-methyl-4-(8-methylnaphthalen-1-yl)-1,4-dihydropyrano[2,3-c]pyrazole-5-carbonitrile.


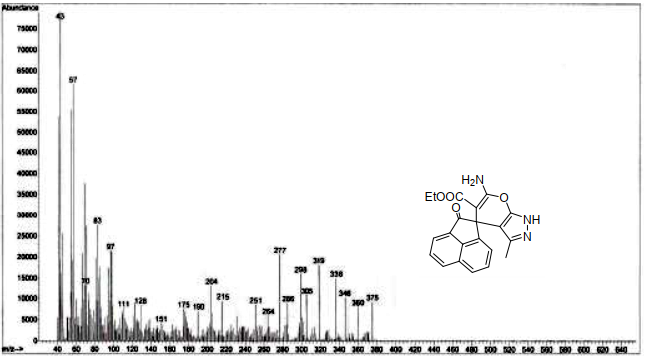


**Figure S44.** Mass spectrum of ethyl 6-amino-4-formyl-3-methyl-4-(8-methylnaphthalen-1-yl)-1,4-dihydropyrano[2,3-c]pyrazole-5-carboxylate.


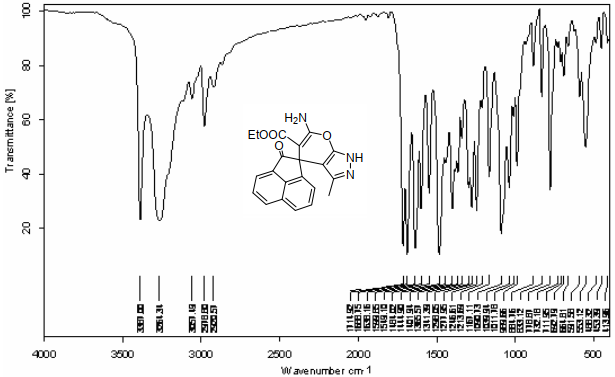


**Figure S45.** FTIR spectrum of ethyl 6-amino-4-formyl-3-methyl-4-(8-methylnaphthalen-1-yl)-1,4-dihydropyrano[2,3-c]pyrazole-5-carboxylate.


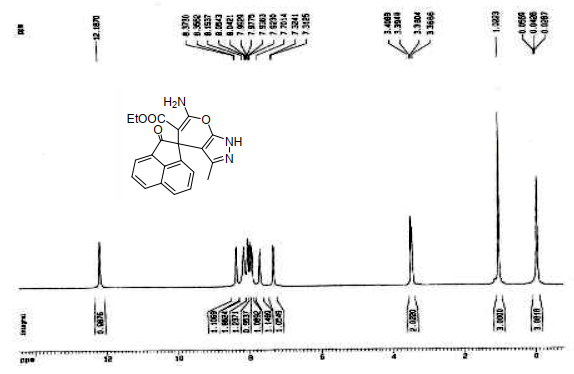


**Figure S46.** ^1^H NMR spectrum of ethyl 6-amino-4-formyl-3-methyl-4-(8-methylnaphthalen-1-yl)-1,4-dihydropyrano[2,3-c]pyrazole-5-carboxylate.


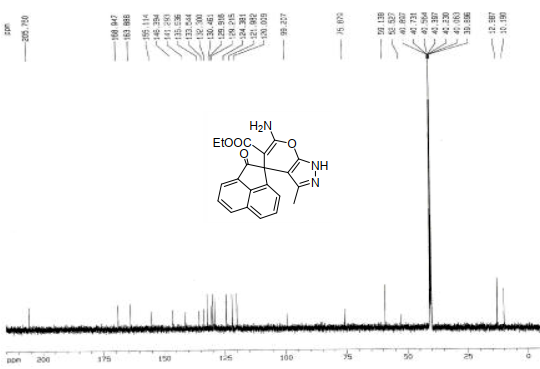


**Figure S47.** ^13^C NMR spectrum of ethyl 6-amino-4-formyl-3-methyl-4-(8-methylnaphthalen-1-yl)-1,4-dihydropyrano[2,3-c]pyrazole-5-carboxylate.
